# Electron‐Sponge Nature of Polyoxometalates for Next‐Generation Electrocatalytic Water Splitting and Nonvolatile Neuromorphic Devices

**DOI:** 10.1002/advs.202304120

**Published:** 2023-11-29

**Authors:** Waqar Ahmad, Nisar Ahmad, Kun Wang, Sumaira Aftab, Yunpeng Hou, Zhengwei Wan, Bei‐Bei Yan, Zhao Pan, Huai‐Ling Gao, Chen Peung, Yang Junke, Chengdu Liang, Zhihui Lu, Wenjun Yan, Min Ling

**Affiliations:** ^1^ Division of New Energy Materials Institute of Zhejiang University‐Quzhou Quzhou 324000 China; ^2^ College of Chemical and Biological Engineering Zhejiang University Hangzhou 310058 China; ^3^ School of Microelectronics University of Science and Technology of China Hefei 230026 China; ^4^ CAS Key Laboratory of Mechanical Behavior and Design of Materials Department of Modern Mechanics CAS Center for Excellence in Complex System Mechanics University of Science and Technology of China Hefei 230027 China; ^5^ School of Automation Hangzhou Dianzi University Hangzhou 310018 China

**Keywords:** electrocatalysts, electron‐sponges, multiple‐electron uptake/release, neuromorphic devices, polyoxometalates

## Abstract

Designing next‐generation molecular devices typically necessitates plentiful oxygen‐bearing sites to facilitate multiple‐electron transfers. However, the theoretical limits of existing materials for energy conversion and information storage devices make it inevitable to hunt for new competitors. Polyoxometalates (POMs), a unique class of metal‐oxide clusters, have been investigated exponentially due to their structural diversity and tunable redox properties. POMs behave as electron‐sponges owing to their intrinsic ability of reversible uptake‐release of multiple electrons. In this review, numerous POM‐frameworks together with desired features of a contender material and inherited properties of POMs are systematically discussed to demonstrate how and why the electron‐sponge‐like nature of POMs is beneficial to design next‐generation water oxidation/reduction electrocatalysts, and neuromorphic nonvolatile resistance‐switching random‐access memory devices. The aim is to converge the attention of scientists who are working separately on electrocatalysts and memory devices, on a point that, although the application types are different, they all hunt for a material that could exhibit electron‐sponge‐like feature to realize boosted performances and thus, encouraging the scientists of two completely different fields to explore POMs as imperious contenders to design next‐generation nanodevices. Finally, challenges and promising prospects in this research field are also highlighted.

## Graphical abstract

Ideally, the development of future nanodevices requires a material that could exhibit multiple and reversible uptake/release of electrons. tStructural diversity and electron‐sponge‐like nature of polyoxometalates make them imperious contenders in designing next‐generation electrocatalytic water splitting and nonvolatile neuromorphic devices.

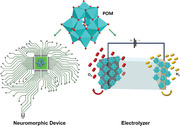

## 1 Introduction

To meet the compactness of the future world, great efforts have been devoted towards energy conversion/storage and information storage technologies, such as water splitting process, and molecular electronic (memory) devices, respectively.^[^ ^1^ ^]^ Importantly, transition metal oxides (TMOs) have been explored as viable materials for electrodes and memristors, owing to the advantages of their relatively high performance, and unique ability to undergo a variety of modifications such as morphology controlling, defect engineering, and material composite, to allow the optimization of their electronic properties.^[^ ^1a^ ^]^ It is noted that the performance of energy conversion (water splitting) devices mainly depends on oxygen evolution reaction (OER), and hydrogen evolution reaction (HER),^[^ ^2^ ^]^ where OER determines the feasibility of energy technologies owing to its sluggish redox kinetics which requires highly active electrocatalysts.^[^ ^3^ ^]^ Generally, precious metal‐, such as Pt, Ru, and Ir, based oxides have been known to demonstrate good electroactivity.^[^ ^4^ ^]^ However, limited by their high cost, poor durability, and low reserves, precious metal materials are difficult to attain widespread commercialization.^[^ ^5^ ^]^ In contrast, earth‐abundant transition metal (TM)‐based electrocatalysts including oxides, carbides, phosphides, and sulfides, are regarded as potential alternatives to noble ones.^[^ ^6^ ^]^ However, their unsatisfied performances owing to lack of abundant redox‐active centers,^[^ ^7^ ^]^ and poor stability under drastic conditions, strictly hinder their industrial applications.^[^ ^1^, ^8^ ^]^ Similarly, the energy storage in rechargeable batteries and electrochemical capacitors which relies on the reversible redox reactions on electrode materials,^[^ ^9^ ^]^ requires a new material that could reveal abundant redox states to enable multi‐electron charge transfer.^[^ ^10^ ^]^

In addition, the development of next‐generation memory devices requires highly effective and durable materials, but as Moore's law^[^ ^11^ ^]^ reaches its physical limit, the gap between conventional and required computing technologies has been increased exponentially.^[^ ^11^, ^12^ ^]^ The current state‐of‐the‐art memory devices are suffering the gigantic challenge of significant decline in computing efficiency as a discrepancy between the data handling speeds of processors and memories, referred to as the “memory wall,” continues to enlarge.^[^ ^13^ ^]^ Human brains possess a complex neural network (≈10^11^ neurons and 10^15^ synapses) for the processing of memory, learning, and other related informations,^[^ ^14^ ^]^ making it impossible for conventional computers to compete with human brain.^[^ ^12b^ ^]^ Numerous efforts have been devoted to emulate the neuromorphic operations in various emerging electronics, such as synaptic memory devices which involve on‐chip data processing, but low operational speed and high‐power consumption during the charging and discharging of the large metallic interconnect bus,^[^ ^15^ ^]^ posing a serious challenge of inherent trade‐off between synaptic characteristics and the necessity for CMOS‐compatible materials and processes.^[^ ^16^ ^]^ In order to mitigate the challenges associated with the conventional microelectronic memories, neuromorphic computing devices, comprising of TMO‐based redox‐induced resistance switching random access memories (RRAMs), are considered as the next‐generation memory devices,^[^ ^17^ ^]^ owing to their characteristics of reversible, and high‐speed drifting of oxygen ions, known as conductive filaments (CFs), through an electric‐field‐induced redox process to exhibit the resistance change.^[^ ^17^, ^18^ ^]^ Although, the first reports of resistive switching phenomena date back to the 1960s,^[^ ^19^ ^]^ but since late 1990s, the research in this field has gained ever increasing attentions.^[^ ^20^, ^119^ ^]^ To date, various TMOs‐based memristors (binary metal oxides (HfO_x_, TaO_x_, and NiO and etc.), ternary metal oxides LaAlO_3_, SrZrO_3_, etc.), chalcogenides such as GeS_x_, nitrides (SiN), polymers, and variety of 2D layered materials^[^ ^20^, ^21^ ^]^ have been explored to achieve the superior energy efficiency of the human brain (10 Watts),^[^ ^22^ ^]^ consuming 5 orders of magnitude lower than supercomputers.^[^ ^20^ ^]^ However, the existing systems remained unsuccessful to exhibit desired performance owing to high power consumption, large size (> 100 µm), low mechanical strength, poor reliability and thermal stability, less flexibility, and weak optical properties.^[^ ^23^ ^]^

In view of aforementioned challenges, due to the theoretical limits of existing materials^[^ ^10^, ^24^ ^]^ towards energy conversion and information storage applications, a unique class of redox‐active nanosized clusters known as polyoxometalates (POMs), consisting of multiple metal‐oxide ions linked together by oxygen atoms to form three‐dimensional (3D) framework, has gained substantial importance towards energy conversion and information storage devices,^[^ ^25^ ^]^ owing to its unique structural‐framework, size, geometry, and adjustable redox properties which enable POMs to display unparalleled physical and chemical properties compared to TMOs.^[^ ^26^ ^]^ This underpins the motivation to investigate the POMs for energy materials and molecular electronic (neuromorphic) devices.

This review presents a comprehensive and systematic research progress of POMs in the frontier of energy conversion (water oxidation and reduction electrocatalysts), and information storage (nonvolatile neuromorphic) devices, in order to converge the attention of scientists who are working separately on electrocatalysts and memory devices, on a point that, although the application types are different (namely electrocatalysts and memory devices), they all hunt for a material that could exhibit better electron uptake/release process, electron‐sponge‐like feature, to realize boosted performances. In this context, POMs which have the inherited ability to withstand multiple uptake/release of electrons without undergoing architectural deterioration, are showing promising performances as electrocatalysts and memory devices, encouraging the scientists of two completely different fields to explore POMs as imperious contenders to design next‐generation nanodevices. Last but not least, outlook and perspectives are proposed for future advances to design innovative POM‐based devices with tunable redox features.

## 2 Structural Framework of POMs

**Figure 1:**
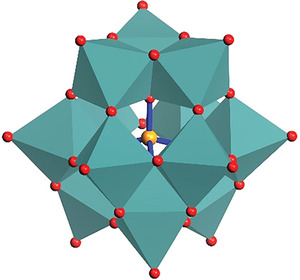
Structural Framework of a Keggin‐type POM. Keggin ([XM_12_O_40_]^n‐^ or XM_12_) structure, where orange ball shows heteroatoms (X = heteroatoms (P, Si, Al); red balls exhibit oxygen atoms; and dark cyan octahedra show transition metals (M).

POMs present thermodynamically stable frameworks with abilities to maintain their characteristic identities in aqueous, organic media or ionic crystals. Their versatile nature, in terms of redox chemistry, structure flexibility, charge distribution, and size range, have attracted interest for the design and development of several multi‐dimensional POM‐architectures.^[^ ^27^ ^]^ POMs comprise some specific arrangements of edge‐sharing and corner‐sharing metal‐oxygen polyhedra MO*_x_* (M = W, Mo, V, and so on; *x* = 5, 6)^[^ ^28^ ^]^ which contains early transition metals in their high oxidation state, usually *d* ^0^ or *d* ^1^ electronic configuration (**Figure** **1**), which can also be substituted with other transition metals (3*d*, 4*d*, 5*d)* to achieve desired metal‐to‐metal interactions, degree of reduction and extent of protonation to apply for a particular field of application.^[^ ^27^, ^29^ ^]^ Thus, the redox activity of POMs arises from the empty and nonbonding d orbitals, *d* ^0^ or *d* ^1^, of the metal centers.^[^ ^30^ ^]^

The abundant availability of oxygen atoms on the surface of POM's framework ensures a highly negative charge on the surface, and hence POMs have the ability to donate a number of electrons to an electron acceptor and this property unambiguously introduces POMs as soft base. The polyhedra of POM's frameworks possess transition metals at the highest oxidation state which allows them to accept many electrons and thus, POMs behave as Lewis acids. Similarly, under different environments, POM can also behave as a Lewis base. Collectively, POMs are regarded as electron‐sponges on account of their ability to donate and accept multiple electrons and therefore project them as a highly redox active class of metal‐oxygen clusters^[^ ^31^ ^]^. Moreover, POM's properties can also be tuned with a variety of counter‐cations. The distinctive interactions of POM‐anions with counter‐cations can dramatically change their electronic properties. Similarly, acidity, basicity, and oxidative nature of POMs can be effectively tuned to an anticipated level.^[^ ^32^ ^]^

The electron‐sponge nature of POMs causes the delocalization of accepted d‐electrons over the 3D framework, consequently modulating the electronic (d‐band) structure correspond to the Fermi level (*E*_F_) which leads to improved electrical conductivity on one hand and optimized adsorption energy between the reaction (OER/HER) intermediates and active sites on the other hand, thus facilitates the reactive kinetics of water oxidation and reduction reactions.^[^ ^33^ ^]^ Similarly, multielectron uptake/release and corresponding delocalization of d‐electrons over a metal framework renders a system imperious contender for application in electronic devices.^[^ ^34^ ^]^ POMs reveal many discrete redox states within a confined potential range, which confirms their ability for multibit data storage and the long‐term stability of the programming/erasing cycles.^[^ ^35^ ^]^

**Figure 2:**
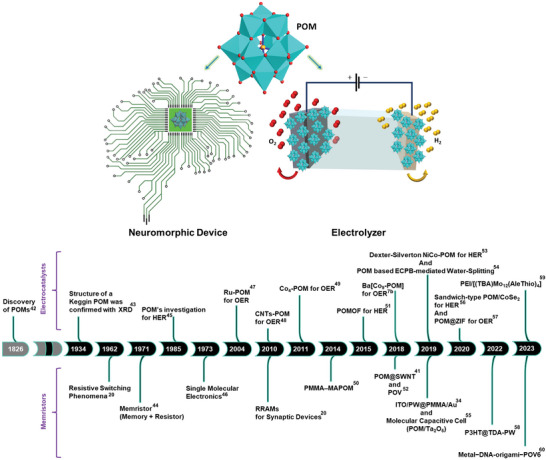
Conceptual illustration of POM's competency for electrocatalytic and neuromorphic devices. A timeline (bottom) is showing some of the major breakthroughs in the area of POM‐based OER/HER electrocatalysts, and memristor technology to POM‐based neuromorphic memory devices.

The design and properties of POMs can be tuned at atomic as well as molecular levels and hence, the versatility of nature and diversity of structural framework make POMs attractive candidates in terms of diverse applications, including medicine,^[^ ^36^ ^]^ magnetism,^[^ ^37^ ^]^ catalysis,^[^ ^38^ ^]^ material sciences,^[^ ^39^ ^]^ electrochemistry^[^ ^40^ ^]^ and neuromorphic systems.^[^ ^34^, ^41^ ^]^ However, this review presents a comprehensive research progress of POMs to converge attention on how and why the electron‐sponge nature of POMs is beneficial to designing next‐generation energy conversion, and information storage devices, as shown in the conceptual illustration (**Figure** **2**).

### 2.1 Structural Types of POMs and their 3D Frameworks

POMs incorporate transition metals (M) (M = W^6+^, Mo^6+^, V^5+^, Nb^5+^, Ta^5+^), regarded as addenda atoms, to develop polyhedra of their architectures. Else than MO_6_ octahedron, POMs ‐framework also includes other geometries of metal oxides such as tetrahedral MO_4_ and trigonal bipyramidal or square pyramidal MO_5_. Such metal‐oxide geometries intend to push addenda atoms away from the central heteroatom towards the vertices that level the surface of polyoxoanion. Hence, POM architectures of different sizes, shapes, and chemical compositions are constructed to obtain extraordinary properties. Numerous POMs have been developed ever since their discovery, mainly they are categorized into three classes.

#### 2.1.1 Heterpolyanions

**Figure 3:**
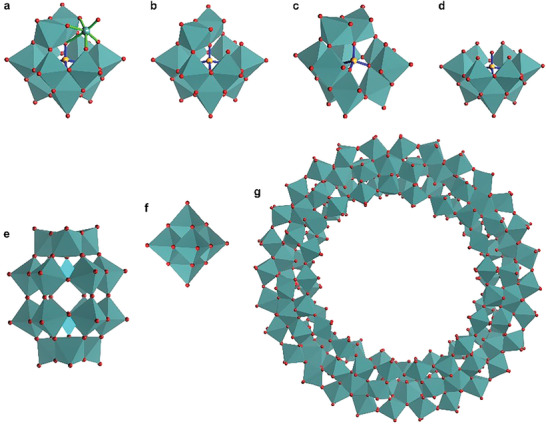
Structural frameworks of different types of POMs. a) Keggin (XM_12_) structure, where orange ball shows heteroatoms (X = heteroatoms (P, Si, Al) of the central tetrahedral core; red balls exhibit oxygen atoms; and dark cyan ball and octahedra show transition metal (addenda) atoms (M) (M = W^6+^, Mo^6+^, V^5+^, Nb^5+^, Ta^5+^). b) Mono‐lacunary ([XM_11_O_39_]^n−^ or XM_11_) c) di‐lacunary ([XM_10_O_38_]^n−^ or XM_10_) and d) tri‐lacunary ([XM_9_O_34_]^n‐^ or XM_9_) Keggin structures which are obtained with the removal of one, two and three MO_6_ octahedra, respectively, from the parent Keggin structure shown in Figure 3a. e) Wells‐Dawson [X_2_M_18_O_62_]^n‐^ structure. f) Lindqvist [M_6_O_19_]^n−^ structure. g) Wheel‐shaped [Mo_154_] cluster.

**Figure 4:**
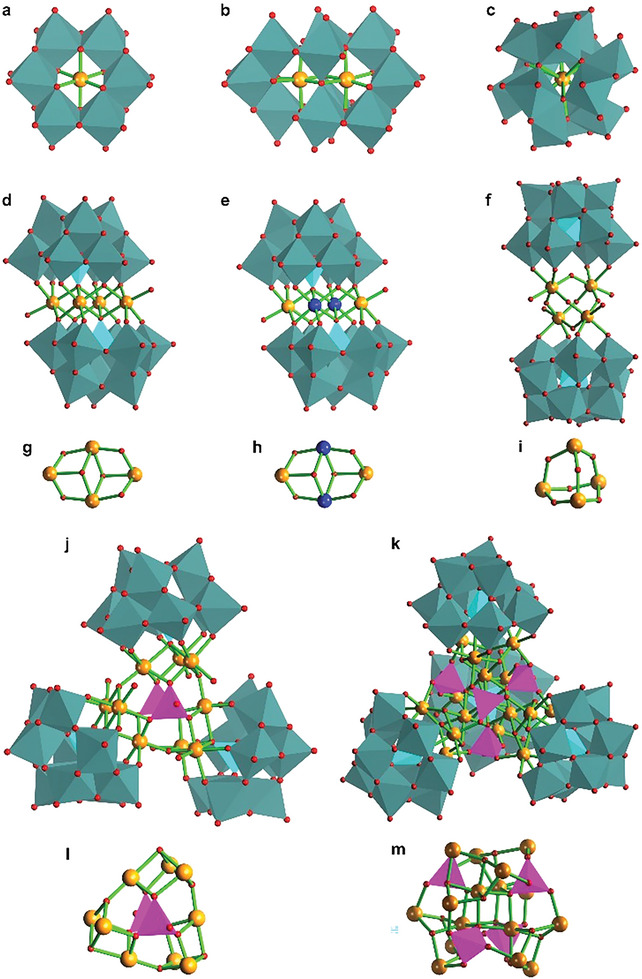
Different POM‐structures with central active‐cores to exhibit tunable redox properties. a) Anderson POM structure. b) Co_2_Mo_10_ structure. c) Dexter‐Silverton structure. d) Sandwich‐type POM with a “cubane” of four similar atoms. e) Sandwich‐type POM with a “cubane” of four different atoms. f) Sandwich‐type polyoxotungstate cluster with a central core of tetra‐ruthenium oxide [Ru_4_O_4_], [Ru_4_(*µ*‐O)_4_(*µ*‐OH)_2_(H_2_O)_4_](*γ*‐SiW_10_O_36_)_2_]^10−^. g) Electroactive tetra‐cobalt core Co_4_O_4_ “cubane” which is usually sandwiched between two oxidatively resistant tri‐lacunary units such as [PW_9_O_34_]^9−^, Wheel‐shaped [Mo_154_] cluster. Similarly, another h) electroactive core but with different atoms. i) Electroactive [Ru_4_O_4_] core, which is stabilized with two units of (*γ*‐SiW_10_O_36_)_2_]^10−^ to make a complete sandwich structure as shown in (f). j) Nona‐cobalt architecture Co_9_(H_2_O)_6_(OH)_3_(HPO_4_)_2_(PW_9_O_34_)_3_^16−^ ( = Co_9_‐POM or Co_9_). k) [Co_x_Fe_4‒x_(OH)_3_ Fe_4‐x_(OH)_3_ (PO_4_)]_4_ core surrounded with four (SiW_9_O_34_)_4_]^n−^ units. l) Central core of Co_9_, where orange balls represent cobalt atoms while pink tetrahedrons show phosphorus atoms. m) Active cobalt‐iron core of [(Co_3_Fe(OH)_3_PO_4_)_4_(SiW_9_O_34_)_4_]^28−^.

These are the most explored polyoxoanions owing to the presence of two different types of atoms, where one type of atom regarded as “addenda” atoms exit in large numbers to develop outer octahedra, whereas the other type of atoms known as “heteroatoms” occur comparatively in small number, to compose inner tetrahedral core. Keggin and Wells‐Dawson are the two main frameworks (**Figure** **3**a,e) of this subclass of polyoxometalates. Keggin structure holds general chemical formula [XM_12_O_40_]^n−^ (where X = heteroatoms (P, Si, Al) to make tetrahedral central core; M = addenda atoms (W, Mo,V) to make polyhedra around the tetrahedral core).^[^ ^61^ ^]^ More than 60 other transition metal atoms and nonmetals can be incorporated as heteroatoms. Keggin [XM_12_O_40_]^n‐^ and Wells‐Dawson structure [X_2_M_18_O_62_]^n−^ both anions have similar types of constituent elements but different numbers of elements. A huge number of derivatives of Keggin and Wells‐Dawson structures have been exploited owing to attractive catalytic properties. Their derivates, known as lacunary framework, are obtained on the removal of one or more polyhedrons (Figure 3b–d) from the parent structures. Lacunary derivates are also considered as efficient candidates for catalytic applications.^[^ ^62^ ^]^ Similarly, numerous POM‐frameworks have been explored which possess a variety of central active‐cores to exhibit tunable redox properties (**Figure** **4**).

#### 2.1.2 Isopolyanions

This class comprises polyoxoanions that are constituted of only one type of high‐valent metals from Group 5 and Group 6 without any central heteroatom. Lindqvist [M_6_O_19_]^n−^ structure (Figure 3f) and their derivatives are among the popular members of this class. The Lindqvist polyoxoanions mostly comprise 4d or 5d metal atoms, somewhere a mixture of 5d and 6d transition metals constitute their frameworks. Different polyhedra in the Lindqvist framework share oxygen atoms at their edges to make a compact structure. Lindqvist POMs, host very interesting properties owing to the highly basic nature of surface oxygen atoms and strong charges on the surfaces, introducing them as robust building blocks.^[^ ^63^ ^]^

#### 2.1.3 Polymolybdate Clusters

In 1995, polymolybdate anion was synthesized and characterized as a mixed valence wheel‐shaped [Mo_154_] cluster.^[^ ^64^ ^]^ In the past couple of years, abundant efforts have been made on high‐nuclearity oxo‐clusters to develop highly symmetric ring‐shaped polymolybdate anionic clusters.^[^ ^65^ ^]^ This class has a strong capability to link different ligands to organize polyoxoanionic clusters into well‐ordered arrangements including mesoporous systems. Therefore, giant polymolybdates (Figure 3g) have emerged as the most exciting class of POM chemistry due to multiple applications in the field of nanotechnology.^[^ ^65a^ ^]^

Unlike the general metal oxides, molecular POMs offer multiple reversible electron transfers, which are responsible for remarkable chemical properties. Thereby, protonated structures of POMs have been explored as stronger and less corrosive acids than the mineral acids HCl and H_2_SO_4_.^[^ ^61^, ^66^ ^]^ Some mixed‐valence and reduced POMs present a blue color, which is known as “heteropolyblue”. Thus, on the bases of their redox and color features, POM frameworks have also been explored for numerous spectrophotochemical determination methods.^[^ ^67^ ^]^

### 2.2 Physicochemical Properties of POMs

POMs exhibit unmatched physicochemical properties which make them imperious contenders to design next‐generation molecular devices.^[^ ^68^ ^]^

The main physicochemical properties of POMs include:

1. Variety of size range.^[^ ^64^ ^]^
2. Remarkable thermal stability.^[^ ^38^, ^66^, ^69^ ^]^
3. Non‐volatile and non‐toxic in nature with high solubility in oxygenated solvents such as water, ether, ethanol, acetone, etc.^[^ ^70^ ^]^
4. A number of surfactants can be incorporated to design hybrid materials.^[^ ^71^ ^]^
5. Chemical composition of POM frameworks can be easily adjusted to achieve prejudice redox properties for a particular purpose.^[^ ^72^ ^]^
6. Ability to withstand multielectron reversible uptake and release without architectural degradation.^[^ ^32c^ ^]^ Subsequently, such accepted electrons delocalize throughout their architecture, a property that renders the application of POMs in electronic devices.^[^ ^34^ ^]^

## 3 Electron‐Sponge Nature of POMs

Electron‐sponge nature of POMs is associated with the reversible or pseudoreversible redox activity of POMs which, in turn, arises from the empty and nonbonding orbitals of metal‐atoms of POM's framework.^[^ ^30^ ^]^ After the reduction process, electrons uptakes, POMs frameworks possess mixed‐valence metal centers where accepted electrons, referred as blue electrons,^[^ ^73^ ^]^ either localize on individual metal ions or delocalize over the whole framework.^[^ ^34^ ^]^ Such d‐electron delocalization can occur in the electronic ground state or thermally induced electron hopping involving π‐bonding of O‐bridge from a metal (M) of low oxidation state to next of high oxidation state (M^V^ −O−M^VI^),^[^ ^73^ ^]^ which is essential for electrocatalysis and multi‐bit storage cell. Under the influence of an external electric potential, metal atoms of POMs, redox‐active centers, accept electrons, and undergo stepwise reduction process (e.g., vanadium V^v^ to V^iv^), causing an increase in the electrical conductance.^[^ ^74^ ^]^ Furthermore, the delocalized d‐electrons modulate the d‐band center (*ε*_d_) near the Fermi level (*E*_F_) and thus perform *E*_F_ engineering in POM frameworks,^[^ ^33a,b^ ^]^ which, in turn, regulates (i) the adsorption strength of intermediates for electrocatalytic reactions,^[^ ^33a^ ^]^ and (ii) the electron transfer from the metal electrodes to the lowest unoccupied molecular orbital (LUMO) of POMs for resistive switching memory devices.^[^ ^75^ ^]^ In this way, the electron sponge nature of POMs enables potential‐induced variation in the electronic structures towards high‐performance electrocatalysis and multibit data storage.

In order to characterize and better understand the electron sponge nature of POMs, a blend of analytical investigations, including cyclic voltammetry (CV), ultraviolet‐visible absorption spectroscopy (UV–vis), Fourier transform infrared spectroscopy (FT‐IR), small‐angle X‐ray scattering (SAXS), electron paramagnetic resonance spectroscopies (EPR), and theoretical calculations based on density functional theory (DFT) are performed to explore the underlying mechanisms of redox processes of POMs.

**Figure 5:**
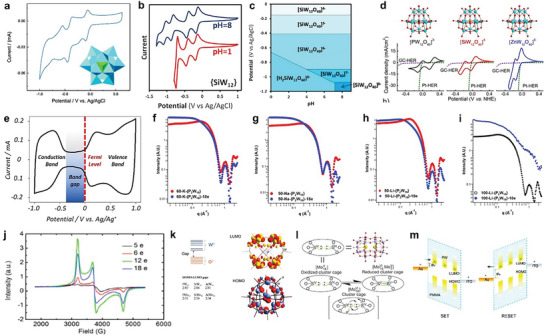
Experimental and theoretical studies to estimate electron uptake/release process in POMs. a) CV result demonstrates the reversible redox behavior of POMs. Reproduced with permission.^[^ ^34^ ^]^ Copyright 2019, Royal Society of Chemistry. b) Cyclic voltammograms of the Si‐POM ([SiW_12_O_40_]^n−^ or SiW_12_) at pH 1 and 8. c) Potential−pH diagram of the [SiW_12_O_40_]^n−^. Reproduced with permission.^[^ ^77^ ^]^ Copyright 2022, American Chemical Society. d) Crystal structure of different Keggin and the corresponding CVs on GCE at 50 m ^−1^s in 100 mM of H_n_X_12_WO_40_ (X = P^5+^, Si^4+^, and Zn^2+^). Reproduced with permission.^[^ ^53^ ^]^ Copyright 2019, John Wiley and Sons. e) Dumbbell shaped CV with key features labeled on it. Reproduced with permission.^[^ ^76^ ^]^ Copyright 2022, John Wiley and Sons. SAXS spectra of f) fully oxidized and fully reduced of K‐P_2_W_18_; (g) Na‐P_2_W_18_ (h) Li‐P_2_W_18_. At similar concentrations for direct comparison; i) Li‐P_2_W_18_ (fully oxidized and fully reduced) at 100 mMolar, showing the large aggregates upon reduction. j) EPR results of Li‐P_2_W_18_ at different reduction states corresponding to the signals for 5−18 electron‐reduction. Reproduced with permission.^[^ ^78^ ^]^ Copyright 2022, American Chemical Society. k) Schematic molecular orbital diagram, HOMO–LUMO energy gaps (in eV), and 3D display of one of the two doubly degenerate components of the LUMO (composed of 73% of d‐metal orbitals) and HOMO (composed of 95% p–oxygen orbitals), the fully oxidized Keggin POM anion. Reproduced with permission.^[^ ^79^ ^]^ Copyright 2001, American Chemical Society. l) The reversible formation of S−S bond and the subsequent electronic redistribution within the structural framework of a POM. Reproduced with permission.^[^ ^80^ ^]^ Copyright 2021, John Wiley and Sons. m) Schematic demonstration of modulation of an interface barrier during the SET/RESET process. Reproduced with permission.^[^ ^34^ ^]^ Copyright 2019, Royal Society of Chemistry.

**Figure** **5**a shows the CV analysis of Keggin‐POM for a resistive switching memory device, where the emergence of reducing and corresponding oxidizing peaks confirms the uptake and release of electron, respectively. Thus, CV study provides the evidence of reversible redox behavior of POMs.^[^ ^34^ ^]^ The electron sponge nature of POMs is also largely influenced by a change in the proton concentrations. Generally, under a basic environment, a redox wave which describes a one‐electron transfer process exists distinctly as a single peak. In contrast, under an acidic environment, the monoelectronic wave merges into a two‐electron wave owing to the incorporation of two protons. Figure 5b, shows that the fourth monoelectronic wave merges with the third monoelectronic wave and makes a single large wave, describing a two‐electron reduction coupled with two protons (α‐[SiW_12_O_40_]^6−^ + 2e^−^ + 2H^+^ → α‐[H_2_SiW_12_O_40_]^6−^) (Figure 5b,c). Such behavior of POMs makes them attractive for energy devices. The CV results shown in Figure 5d reveal the effect of heteroatoms (X = P^5+^, Si^4+^, and Zn^2+^) on the redox chemistry of POMs corresponding to the HER voltage range. Interestingly, the prominent ionic charge of anionic clusters causes a negative shift in the midpoint potential of the first redox wave.^[^ ^54^ ^]^ The charge transfer in nanocomposites can be estimated with an appropriate comparison of characteristic “dumb‐bell” shape CV and the electronic density of states (DOS) studies.^[^ ^76^ ^]^ CV analysis of POMs can identify the positions of the conduction and valence bands of host materials and the LUMOs of the POMs (Figure 5e). Generally, the reductions of POMs occur at more positive potential than the top of the valence band of host materials such as carbon nanotubes, leading to charge transfer from host material to the lower‐lying LUMOs of the POMs, confirming electrostatic binding of POMs and the oxidization of host material.^[^ ^76^, ^77^ ^]^

Valence states of POMs can be characterized with UV–vis absorption spectroscopy. It provides information about the intervalence charge‐transfer and the valence state of POMs, which is judged according to the change of absorbance.^[^ ^82^ ^]^ FT‐IR reveals the structural stability of POMs. Generally, FT‐IR analysis of a reduced and oxidized POM reveals no prominent changes in the spectra. However, FT‐IR analysis before and after the electrochemical charge–discharge cycles, estimates the chemical stability of POMs.^[^ ^83^ ^]^ Furthermore, to differentiate oxidized and reduced POMs, SAXS analysis is performed. The emergence of the coulomb peak represents the homogeneous dispersion of species, while the absence of coulomb peak indicates the aggregation of species.^[^ ^79^ ^]^ The Guinier region (*q*> 0.2 Å^−1^) of SAXS curves provides information about the size of the dissolved particles.^[^ ^84^ ^]^ Comparative analysis of SAXS curves of oxidized and reduced POMs of different cations (K^+^, Na^+^, and Li^+^) shows that the coulomb peak of K‐P_2_W_18_‐18e and Na‐P_2_W_18_‐18e are strongly shielded while that of Li‐P_2_W_18_‐18e is weakly shielded, in the Guinier region (Figure 5f‐i). Despite the same positive charge, the small ionic radius of Li^+^ compared with that of K^+^ and Na^+^, owns a larger hydration radius, which decreases the ability to neutralize highly negatively charged POM anions, therefore exhibiting a partial shielding of the coulomb peak for Li‐P_2_W_18_‐18e. The weak shielding of the coulomb peak in the Guinier region suggests a bigger size of particles which is associated with the high reduction of POMs and subsequent aggregation of POM clusters.^[^ ^79^ ^]^ EPR analysis characterizes the degrees of reduction in POMs with an increase in the intensity of measured signals corresponding to the number of reducing electrons. It has been reported that when the number of accepted electrons increased from 5 to 12 in a POM (Li‐P_2_W_18_), the intensity of EPR signals showed a continuous increase, which was attributed to the addition of protons and subsequent transformation of W = O to W^5+^‐OH units (Figure 5j). However, the signal intensity showed a decline with the acceptance of more electrons from 12 to 18, which was comparable to the number of accepted electrons in the POM.^[^ ^79^ ^]^

DFT investigations have revealed that POM anions ([XM_12_O_40_]^n−^, (M = W, Mo; X = Co^II^, Al^III^, Si^IV^, P^V^) without central paramagnetic ions X possess a ground state configuration which is similar to that of a fully oxidized POM anion and keep a high energy gap between the highest occupied molecular orbital (HOMO), delocalized over oxo‐ligands, and the LUMO, delocalized over the d‐shells of addenda metal atom “M” (M = W, Mo).^[^ ^85^ ^]^ Noticeably, in fully oxidized POM anions, the nature of X has no influence on this energy gap while M strongly influences the energy gap. Therefore, in POMs with M = W, this energy gap is relatively higher than the POMs with M = Mo, which suggests that the lower d‐orbitals in Mo‐based POMs allow relatively easier uptake of electrons. Hence, Mo‐based POM frameworks behave as strong oxidizing agents than the W‐based POMs (Figure 5k).^[^ ^80^ ^]^ Moreover, DFT studies on a pseudo‐Dawson‐type [(SO_3_)_2_Mo_18_O_54_] cluster validate an intramolecular redox reaction that emerged with bond formation between the S‐atoms of two sulfite groups in a POM framework (Figure 5l). To assess a controlled redox behavior, POM clusters were adsorbed on gold surface, and an electric field was applied to activate the redox feature. Under the influence of an applied electric field, [Mo_18_O_54_] oxide shell accepted two electrons and produced a mixed valence state due to the transformation of a fully oxidized [Mo^VI^_18_] to [Mo^VI^_16_Mo^V^_2_]. Intriguingly, this reversible electron transformation was ascribed to the formation of an S−S bond between the (S^IV^O_3_)_2_ groups of two encapsulated pyramids. Consequently, the S−S formation inside the Mo_18_O_54_ cluster shell created a new electron‐rich state inside the bandgap of the (SO_3_)_2_Mo_18_O_54_ cluster. Analytical and theoretical studies have proved that the adsorption of POMs onto a highly polarizable metal surface can accelerate a reversible intramolecular redox process which facilitates electron uptake into the POM shells. Hence, this process reversibly modulates the electronic structure of POMs, suggesting great potential for applications in molecular electronic devices.^[^ ^86^ ^]^ Furthermore, DFT studies have been explored to analyze the state transformation of POMs before and after the electron's acceptance. DFT calculations have demonstrated that the accepted electrons cause variations in the HOMO and LUMO of POMs corresponding to the electron acceptance barrier, which can be related to the redox‐based SET/RESET processes in resistive switching nanodevices. During the SET process, POM clusters accept electrons during the reduction process and subsequently delocalize the charge carriers over the POM clusters to exhibit the conduction mechanism, loading to a resistance switching behavior. In contrast, during the RESET process, the resistance switching from the low resistance state (LRS) (or ON state) to the high resistance state (HRS) (or OFF state) HRS attributes electron extraction and recombination efficiency to increase the charge transportation.^[^ ^34^ ^]^ The SET and RESET processes of a memory device are illustrated in Figure 5m.

## 4 Electrocatalysts for OER and HER

### 4.1 Traditional Materials for Anodic OER and Cathodic HER

Traditional materials for anodic OER include oxides/composites of precious metals such as iridium and ruthenium. These materials reveal a small overpotential for OER, meaning that a low voltage (millivolt) is required to initiate the reaction. However, unavoidable limitations such as low reserves, high cost, and insufficient durability over long‐term operation, have restricted their widespread practical applications.^[^ ^33a^ ^]^ Similarly, for cathodic HER, traditional materials involve platinum group metals (PGMs) which include platinum, palladium, and gold due to their low overpotential and high catalytic activity for the HER. However, their scarcity and high‐cost limit their widespread use in large‐scale H_2_ production.^[^ ^4b^ ^]^ The traditional electrocatalysts may have low overpotentials, which means that they require small energy input to perform electrocatalytic OER and HER but only a few catalysts reveal high selectivity for the targeted formation of H_2_.^[^ ^87^ ^]^ Moreover, the production of PGMs‐based materials for water‐splitting electrodes generates significantly harmful environmental impacts, mainly related to mineral extraction, processing, and disposal.^[^ ^88^ ^]^ To overcome these challenges, low‐cost alternatives such as M‐Xide family (M = nonprecious transition metal (M), X = O, C, N, P, S, Se, Te, B),^[^ ^89^ ^]^ organic frameworks of Ni, Cu, CO, and Zn,^[^ ^90^ ^]^ and variety of dimensional nanomaterials (0D to 3D)/composites catalysts^[^ ^91^ ^]^ have been investigated for water splitting electrodes but their low activity and stability under drastic conditions, mainly hinder large‐scale applications.^[^ ^1^, ^8^ ^]^

Designing robust electrocatalysts to achieve high activity, durability, and affordability requires excellent knowledge of the tuning of the fundamental features such as redox‐properties,^[^ ^92^ ^]^ selectivity,^[^ ^87^ ^]^ and *ε*_d_ modulation^[^ ^33a^ ^]^ of a (catalyst) system, and comprehensive understanding of OER/HER processes.^[^ ^8^ ^]^

### 4.2 Mandatory Features of a Material for OER Electrocatalysis

The development of robust electrocatalysts requires following features incorporated in their structural designs in order to assess highly efficient and durable water oxidation performance.

1. Possessing abundant redox‐active centers to demonstrate proton‐coupled‐electron transfer (PCET) prior to water oxidation. A variety of engineering efforts are directed to develop a PCET feature within a system, a millstone, in designing robust OER electrocatalysts. POM architectures incorporate multiple redox‐active transition metals that can facilitate PCET prior to water oxidation. The presence of multiple redox‐active centers can easily tackle four‐electron transfer process, a bottle‐neck for the water oxidation reaction. POM can comprise four or somewhere up to nine redox‐active centers in their frameworks.^[^ ^7a,b^ ^]^
2. Revealing sites to coordinate aquo ligands. The unique architecture of POMs offers abundantly accessible sites to bind aqua ligands.^[^ ^93^ ^]^
3. The design of a robust water oxidation catalyst demands to fulfill the requirement of Bronsted acid‐base chemistry, as Bronsted bases play a vital role in the transfer of protons from water‐substrate. POM frameworks offer multiple terminal and bridging oxo‐ligands as possible proton binding sites adjacent to the redox active centers (cubane cores) to facilitate the proton‐coupled electron transfer process.^[^ ^94^ ^]^
4. Ability to allow the modulation of its electronic structures. As POM's frameworks comprise of high‐valent metals (such as W) and highly electronegative non‐metals elements (such as P), where substitution of an element of different electronegatively can significantly regulate the *ε*_d_ which, in turn, modulates the adsorption strengths of reaction intermediates during variety of electrocatalytic processes.^[^ ^33a,c^ ^]^
5. The scalability is mandatory for electrocatalysts to ensure commercially viable production, which essentially depends on the cost of raw materials and the complexity involved in the synthesis routes. Therefore, the preparation of precious‐metals‐free catalysts via facile synthesis routes can significantly improve the scalability.^[^ ^95^ ^]^

### 4.3 POM‐Based OER Electrocatalysts

**Figure 6:**
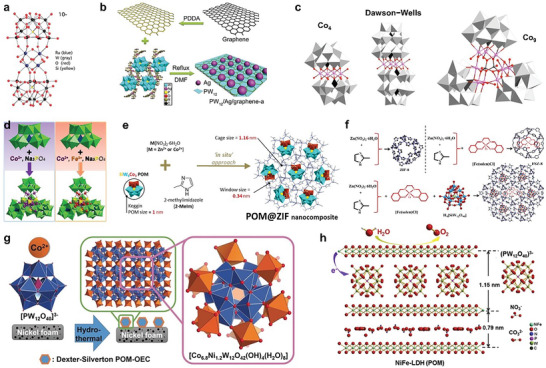
POM‐based electrocatalysts for the OER. a) Tetra‐ruthenium‐functionalized POM [Ru_4_(*µ*‐O)_4_(*µ*‐OH)_2_(H_2_O)_4_](*γ*‐SiW_10_O_36_)_2_]^10−^, where Lacunary units stabilize the whole architecture and establish a Bronsted acid‐base chemistry to provide multiple proton‐transfer sites to facilitate water oxidation. Reproduced with permission.^[^ ^95^ ^]^ Copyright 2013, RSC Publishing. b) Proposed growth mechanism of PW_12_/Ag/graphene composite. Reproduced with permission.^[^ ^98^ ^]^ Copyright 2019, John Wiley and Sons. c) Molecular structure of a sandwich‐type Co_4_ or [Co_4_(H_2_O)_2_(PW_9_O_34_)_2_]^10−^, Dawson‐Wells‐type Co_4_ or [Co_4_(H_2_O)_2_(P_2_W_15_O_56_)_2_]^10−^, and Co_9_ or [Co_9_(H_2_O)_6_(OH)_3_(HPO_4_)_2_(PW_9_O_34_)_3_]^16−^, where WO_6_ = gray octahedra; PO_4_ = black octahedra; Co = pink; P = black; O = red. Reproduced with permission.^[^ ^7a^ ^]^ Copyright 2012, American Chemical Society. d) Synthetic routes to [(Co_x_Fe_4‒x_(OH)_3_ Fe_4‐x_(OH)_3_ (PO_4_))_4_(SiW_9_O_34_)_4_)]^n−^. (*X* = 4, 2, or 1; n = 32, 24, or 28), where green octahedra, blue tetrahedra, green sphere, violet sphere, and yellow spheres represent WO_6_, SiO_4_, PO_4_, O, Co, and P, respectively. Reproduced with permission.^[^ ^106^ ^]^ Copyright 2020, Elsevier. e) POM@ZIF nanocomposites. Reproduced with permission.^[^ ^56^ ^]^ Copyright 2020, American Chemical Society. f) Schematic representation of the syntheses of ZIF‐8 and [Fe(salen)(OH)]@ZIF‐8 (FSZ‐8). Reproduced with permission.^[^ ^109^ ^]^ Copyright 2020, American Chemical Society. g) Dexter–Silverton POM catalyst. Reproduced with permission.^[^ ^111^ ^]^ Copyright 2017, John Wiley and Sons. h) Intercalation of Keggin POM in NiFe LDH. Reproduced with permission.^[^ ^112^ ^]^ Copyright 2020, Elsevier.

For the first time, a di‐ruthenium substituted Zn‐POM, Na_14_[Ru^III^_2_Zn_2_(H_2_O)_2_‐(ZnW_9_O_34_)_2_], was selected for electrochemical water oxidation to produce molecular oxygen in aqueous phosphate buffer at pH 8.0. In this POM framework, two ruthenium and two zinc atoms were sandwiched between two units of Zn‐tungstate. This di‐ruthenium substituted‐POM was prepared from precursor Zn‐POM, Na_12_[WZnZn_2_(H_2_O)_2_(ZnW_9_O_34_)_2_].^[^ ^47^ ^]^ This work mainly highlighted the significance of substituted sandwich core of POM‐framework to generate O_2_. It clearly shows that stabilization of an electroactive core between metal‐oxo units can develop an efficient a highly durable electrocatalyst for water oxidation. Therefore, later efforts on water oxidation catalysts (**Figure** **6**a), tetra‐ruthenium‐functionalized polyoxotungstate cluster [Ru_4_(*µ*‐O)_4_(*µ*‐OH)_2_(H_2_O)_4_](*γ*‐SiW_10_O_36_)_2_]^10−^ (ref. [96]) confirmed that an appropriately sandwiched tetra‐ruthenium oxide, [Ru_4_O_4_], can be applied as highly efficient core to perform water oxidation.^[^ ^97^ ^]^ It also reveals the significance of lacunary POM units, [*γ*‐SiW_10_O_36_]^8−^, in designing an efficient oxo‐framework. Lacunary POM units usually perform as tetradentate oxygen‐donor ligands and help in stabilizing the whole architecture. POM units around the tetra‐ruthenium core help to establish Bronsted acid‐base chemistry and provide multiple proton‐transfer sites, and thus, POM actively plays a vital role during water oxidation.^[^ ^96^, ^98^ ^]^ Two Keggin‐type POM, H_3_[PW_12_O_40_] = PW_12_, containing biimidazole (H_2_biim) complexes, have been used as precursors to synthesize composite catalysts with poly(diallyldimethylammonium chloride) (PDDA)‐modified graphene, as a carrier, in the presence of N,N‐dimethylformamide (DMF) as a reducing agent, through a one‐step synthesis route (Figure 6b). The as‐prepared two composite catalysts revealed superior OER performance neutral medium (540 and 610 mV at 10 mA cm^–2^, with Tafel slope of 190 and 192 mV dec^–1^, respectively) due to a synergistic interfacial charge transfer, which emerged from the interaction of Ag nanoparticles, PW_12_, and graphene. This work gives insight into designing efficient water oxidation catalysts using redox‐active Ag‐based POMs.^[^ ^99^ ^]^ A cobalt‐based POM architecture, [Co_4_(H_2_O)_2_(PW_9_O_34_)_2_]^10−^, where a tetra‐cobalt core Co_4_O_4_ “Co cubane to transfer electrons” was sandwiched between two oxidatively resistant tri‐lacunary phosphotungstate units [PW_9_O_34_]^9−^, was developed as a noble metal independent framework to investigate water oxidation activity.^[^ ^100^ ^]^ There is another sandwich‐type POM architecture, Na_10_[Co_4_(H_2_O)_2_(VW_9_O_34_)_2_]·35H_2_O, where tetra‐cobalt core is sandwiched between two [VW_9_O_34_]^9−^ units. The comparative water oxidation efficiency revealed that Co_4_O_4_ cubane sandwiched between two [VW_9_O_34_]^9−^ fragments outperformed the POM in which Co_4_O_4_ cubane was sandwiched between two [PW_9_O_34_]^9‐^ units.^[^ ^101^ ^]^ It shows that a careful tuning of redox properties of POM units can be achieved with the suitable substitution of heteroatoms of POM architectures.^[^ ^54^ ^]^ In addition, another cobalt‐based POM was explored for water oxidation applications. In which, three tri‐lacunary phosphotungstate segments, [PW_9_O_34_]^9−^, were used to surround a central core, Co_9_(H_2_O)_6_(OH)_3_(HPO_4_)_2_, to establish Co_9_(H_2_O)_6_(OH)_3_(HPO_4_)_2_(PW_9_O_34_)_3_^16−^ ( = Co_9_) (Figure 6c).^[^ ^7a^ ^]^ Experimental evidence reveals that Co_9_‐POM was a robust and durable OER catalyst. Immobilization of electroactive materials into carbon paste (CP) has become a popular approach to explore electrocatalysts for water oxidation. Shan and coworkers entrapped a cobalt‐based monosubstituted Keggin‐type POM, [Hpy]_2_[Co(4,40‐Hbpy)_2_(H_2_O)_2_][SiCoW_11_O_39_], in carbon paste to investigate electrocatalytic water oxidation activity.^[^ ^102^ ^]^ There are some POM architectures that incorporate a transition metal atom as their central atom in the framework such as molybdenum‐based mono‐cobalt [H_6_Co^III^Mo_6_O_24_H_6_]^3−^ and di‐cobalt [H_4_Co^III^_2_Mo_10_O_38_]^6−^ POMs.^[^ ^103^ ^]^ The electrochemical investigation highlights the significance of redox‐active transition metals around the cobalt tetrahedral core. It is suggested that POMs centering a redox‐active transition metal show high and stable activity due to redox‐active and stabilization nature of POM's framework.^[^ ^104^ ^]^

Zeolitic imidazolate framework (ZIF) has been used to encapsulate cobalt‐based Keggin POM. A kegging‐type cobalt POM [CoW_12_O_40_]^6‐^, has been immobilized in ZIF‐8, composed of Zn^2+^, to achieve stable electrocatalyst for water oxidation. The as‐prepared [H_6_CoW_12_O_40_]@ZIF‐8, showed good electrocatalytic water oxidation efficiency at pH 8 in aqueous 0.1 m Na_2_SO_4_ and remained stable after long‐term chronoamperometry study. Furthermore, controlled experiments ruled out the chances of CoO_x_ as active species the electrocatalytic water oxidation process.^[^ ^105^ ^]^ Other than this, new work has been reported on a cobalt‐based POM, [Co_4_(OH)_3_(PO_4_)]_4_(SiW_9_O_34_)_4_]^32−^ where Co_4_O_4_ cubane, analogous to the Mn_3_CaO_4_ cubane of the oxygen‐evolving complex in photosystem II, is surrounded by four tri‐lacunary silicotungstate, [SiW_9_O_34_]^10−^, fragments.^[^ ^106^ ^]^ Moreover, Fe substitution of the parent POM, [Co_4_(OH)_3_(PO_4_)]_4_(SiW_9_O_34_)_4_]^32−^, was carried out to improve catalytic activity for water oxidation in acidic medium (Figure 6d). The prepared barium salt of Fe‐substituted POM, Ba_14_[[FeCo_3_(OH)_3_PO_4_]_4_(SiW_9_O_34_)_4_], revealed enhanced electroactivity in acidic electrolyte as water oxidation catalyst with well‐maintained structural integrity over 2000 cyclic voltammetry (CV) cycles and long‐term durability even after 24 h electrocatalytic investigation. This effort presents a model to study the structure‐property correlations, with the aim of designing robust molecular POM‐based heterogeneous electrocatalysts.^[^ ^107^ ^]^ The development of bidirectional synergic interactions from the guest@host‐type nanostructures synthesis approach, has been assessed to boost OER performance of POM and ZIF‐based electrocatalysts (Figure 6e) in an alkaline medium.^[^ ^57^ ^]^ POMs are capable to withstand to facilitate reversible electron transfers,^[^ ^40^ ^]^ while ZIFs have extended 3D cage‐like frameworks that reveal extraordinary scope for catalytic applications owing to highly ordered microporosity and availability of transition metals in the nodes of their architectures.^[^ ^108^ ^]^ Recently, a cobalt based POM, [SiW_9_Co_3_(H_2_O)_3_O_37_]^10−^ = (SiW_9_Co_3_), was immobilized separately in two ZIFs; ZiF‐8 and ZIF‐67, composed of Zn^2+^ and Co^2+^, respectively, to investigate electrocatalytic efficiency for water oxidation. Insight analyses showed that the SiW_9_Co_3_–ZIF‐67 developed interesting ZIF ↔ POM bidirectional synergy that involved Co‐sites of POM clusters as a result of electron transfer from ZIF‐67 to SiW_9_Co_3_, and POM‐induced more active unsaturated Co‐nodes of the ZIF‐67 architectures. This work presents a new concept to develop highly efficient and durable POM‐based electrocatalysts in the future.^[^ ^57^ ^]^

Various first‐row transition‐metal mononuclear complexes have the ability to undergo oxidation to achieve their highest oxidation state to develop a metal‐oxo intermediate species to accomplish catalytic water oxidation. In a well‐known complex of iron, [Fe^III^(salen)Cl], where changes from Fe^III^ to Fe^IV^,[(Fe^IV^ = O)(salen)]^+^, prior to the catalytic oxidation of organic molecules, but the area of Fe‐salen based electrocatalysts for water oxidation remained unexplored owing to the instability of Iron (III)− salen complexes under oxidizing environments of electrochemical OER.^[^ ^109^ ^]^ A very recent report on POM, Iron (III)− salen, and ZIF‐8 based heterogeneous electrocatalysts revealed a robust electrocatalyst (Figure 6f) for water oxidation in neutral medium. In this work, [Fe^III^(salen)Cl] and silicon‐based POM, H_4_[SiW_12_O_40_], were co‐encapsulated inside the ZIF‐8, FSWZ‐8, via an in situ synthesis approach, where ZIF‐8 performed as a highly porous host, Fe‐salen as the active agent, and POM assisted in charge transport and hence, lowered the electrical resistance of the composite catalyst. This work highlighted the significance of POMs in stabilizing the Fe‐salen species along with the whole composite system in neutral pH. Encapsulation of POM accelerated the synthesis of FSWZ‐8 composite and allowed higher loading of iron‐salen and, most prominently, the presence of molecular POMs inside the cavities as well as on the surface of ZIF‐8, ensuring electrical charge conduction in the ZIF‐8 matrix, which in turn lower the charge‐transfer resistance and subsequently, decrease the required overpotential for OER by greater than 150 mV. This work demonstrated the huge potential to design composites catalysts for the electrochemical applications.^[^ ^110^ ^]^ Similarly, a POM‐supported nickel‐based complex has been reported for heterogeneous water oxidation application in a neutral medium.^[^ ^111^ ^]^ It is found that the firm linkages of first row transition metal complexes to suitable POM not only make the system durable but also robust their heterogeneous electrocatalytic applications where most of the complexes, containing organic agents in their framework, start to deactivate owing to structural degradation under harsh environment.^[^ ^109^ ^]^ This effort provides a glimpse that POM‐supported transition‐metal complexes have great potential to be explored as highly durable and efficient heterogeneous electrocatalysts for water oxidation applications.^[^ ^111^ ^]^

To achieve the goal of high surface area with remarkable conductivity, commercial metallic foams are considered as prime candidates to develop robust electrocatalysts. Streb, Song. and their colleagues have reported their efforts in developing POM‐based water oxidation catalysts based on Keggin or lacunary‐Keggin POMs (Figure 6g).^[^ ^112^, ^114^ ^]^ The prepared electrocatalysts revealed long‐term stability under highly drastic alkaline condition (pH 13). Spherical nanoparticles of a Keggin‐type molybdenum‐based POM [H_3_PMo_12_O_40_], [HPMo]NPs, have been reported to modify a gold working electrode, [HPMo]NPs–Au, to investigate the electrocatalytic water oxidation in a phosphate buffer at neutral pH. POM‐based gold electrode, [HPMo]NPs–Au, revealed outstanding OER electrocatalytic performance at low overpotential 350 mV at ≈1.7 mA cm^−2^ along with remarkable long‐term durability under exhaustive electrolysis conditions.^[^ ^115^ ^]^

Layered double hydroxide (LDH) materials are regarded as potential materials to perform as a suitable alternative to state‐of‐the‐art OER electrocatalysts (RuO_2_ and IrO_2_). However, the electrocatalytic efficiency of LDHs needs to be improved as the bigger size and high thickness of the bulk materials largely limit the number of accessible exposed active sites.^[^ ^116^ ^]^ Exfoliation of bulk‐layered double hydroxides is considered a a decent strategy to improve the catalytic performance of LDH materials.^[^ ^117^ ^]^ The incorporation of POMs has the ability to improve the local electronic structure to boost the charge transport capacity of NiFe‐LDHs. POM‐induced NiFe‐LDH (Figure 6h), prepared via an exfoliation and self‐recombination approach, showed that the height of the intralayer spaces of the LDHs was increased to double, and hence, the electronic structure of the nickel species was also modulated. POM‐based NiFe‐LDH, demonstrated enhanced OER electrocatalytic efficiency in an alkaline medium with a low overpotential of 322 mV@50 mA cm^−2^ and remarkable long‐term durability. This effort inspires how to optimize charge transport capability and improve intrinsic electrocatalytic kinetics of single catalytic active sites to achieve enhanced electrocatalytic activity of LDH materials.^[^ ^113^ ^]^

### 4.4 POM‐Based OER Electrocatalysts versus State‐of‐the‐Art Counterparts

Designing high‐performance OER electrocatalysts is the main bottleneck for molecular hydrogen production from water splitting owing to the sluggish kinetics associated with the complicated transfer of multiple electrons.^[^ ^118^ ^]^ Electrocatalysts used to reduce the energy consumption in these reactions are mainly based on precious metals with high‐costs. The ongoing efforts for low‐cost and efficient electrocatalysts have proven to be difficult; most developed catalysts only operate below 100 mA cm^−2^, which is unsatisfactory for large‐scale production. Another problem in testing these electrocatalysts is the difference between electrolyte concentration, temperature, and pressure of lab‐scale and industrial‐scale cells, which substantially affects the electrolyte conductivity, ion migratory flux, and durability of a catalyst.^[^ ^119^ ^]^ The low‐cost and precious‐metals‐free materials, namely POMs, have huge potential to overcome the aforementioned problems and perform electrocatalysis at high efficiency. The unique structure of POMs anions, comprised of transition metals (e.g., Mo, W, V) and oxygen atoms, leads to abundant redox‐active centers, which is extremely vital for the development of multi‐electron transfer OER processes. The high thermal stability, high sensitivity to electricity, and resistance to oxidative decomposition render POMs promising toward electrocatalytic applications. The distinct redox capability of POMs allows reversible uptake of as much as 30 electrons per POM molecule in the solid state, indicating the applicability of POMs for multi‐electron transfer processes.^[^ ^120^ ^]^ Thus, the electron‐sponge nature of POMs, owing to the redox‐active metal centers of POM's structural framework, determines the electrochemical behavior of POMs in terms of redox‐potential and electrons‐storage, and thus ensures the adequate charge flow which is inevitable for numerous electrochemical/electroanalytical applications.^[^ ^121^ ^]^ Covalent or non‐covalent anchoring of POMs on conductive and high‐surface‐area substrates improves the charge flow for energy devices.^[^ ^122^ ^]^ In addition, as the constituents of POM frameworks are all inorganic (Figure 4, 6) and difficult to be oxidized during the water oxidation process, POMs and their derived composites have been widely used as stable water oxidation catalysts. Therefore, unlike traditional electrocatalysts, POMs not only show remarkably high OER performances under extremely acidic and basic environments but also outperform their commercial counterparts.^[^ ^7^, ^112^ ^]^

### 4.5 Mandatory Features of a Material for HER Electrocatalysis

The development of robust HER electrocatalysts requires the following features incorporated in their designs in order to assess benchmarked performance:

1. Ability to undergo reversible multi‐electron reduction/oxidation without deterioration. POMs can offer multiple electron release and uptake without structural destruction, thus behaving like electron sponges.^[^ ^7c^ ^]^
2. Requires to show electron uptake and release near the thermodynamic potential of HER. POMs have the capability to release and uptake electrons in a potential domain close to the thermodynamic potential of HER.
3. Needs to facilitate the proton transfer process. Versatile POM architectures can undergo multiple electron reduction and has the ability to uptake multiple protons, hence fulfilling the requirement of proton‐coupled electron transfer (PCET).^[^ ^123^ ^]^
4. Ability to maintain a high loading of the active species at the electrode surface (due to its high surface area and morphology (2D or 3D)), and subsequently establish a synergy with conductive support to facilitate multiple electron transfers.

### 4.6 POM‐Based HER Electrocatalysts

**Figure 7:**
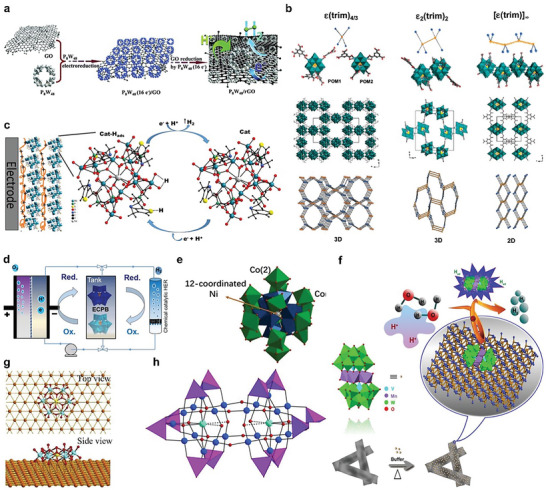
POM‐based electrocatalysts for the HER. a) Scheme of the one‐step electrochemical reduction synthesis of the P_8_W_48_/rGO nanocomposite. Reproduced with permission.^[^ ^122^ ^]^ Copyright 2016, Royal Society of Chemistry. b) POM building blocks of *ε*(trim)_4/3_, *ε*_2_(trim)_2_, and [*ε*(trim)]_∞_, where black lines indicate connections between POMs and trim linkers, while orange lines symbolize condensation reactions between POMs. Reproduced with permission.^[^ ^137^ ^]^ Copyright 2011, American Chemical Society. c) An electrocatalytic cycle for H^+^ reduction at a POM‐based bilayer‐modified electrode surface. Reproduced with permission.^[^ ^58^ ^]^ Copyright 2023, American Chemical Society. d) ECPB‐mediated water‐splitting system and the spontaneous H_2_ production system. Reproduced with permission.^[^ ^53^ ^]^ Copyright 2019, John Wiley and Sons. e) Dexter‐Silverton NiCo‐POM. Color scheme: Ni grey, Co green, W blue, O red. H atoms omitted for clarity. Reproduced with permission.^[^ ^52^ ^]^ Copyright 2019, John Wiley and Sons. f) Schematic illustration of sandwich‐type POM decorated on the CoSe_2_‐nanobelts. Reproduced with permission.^[^ ^55^ ^]^ Copyright 2020, John Wiley and Sons. g) Representation of chemical association simulation of Anderson‐type POM on Cu ^[^ ^111^ ^]^ surface. Reproduced with permission.^[^ ^143^ ^]^ Copyright 2019, Elsevier. h) Double‐cuboid‐shaped [Cu_2_Pd_22_P_12_O_60_(OH)_8_]^20−^. Reproduced with permission.^[^ ^144^ ^]^ Copyright 2011, John Wiley and Sons.

Surface modification of cheap materials can become useful for electrocatalytic applications.^[^ ^124^ ^]^ Immobilization of POMs in 3D configuration increases the basicity of entrapped POMs^[^ ^125^ ^]^ which in turn facilitates the electron uptake and release in a potential domain close to the required thermodynamic potential for efficient catalysis. The confinement of POM in a 3D configuration in a polymer matrix is, generally, regarded as “microenvironment effect”.^[^ ^125^, ^126^ ^]^ Vulcan XC‐72, polyvinylpyridine (PVP) and slightly quaternized polyvinylpyridine (QPVP) have been reported to entrap and finally deposit different POMs, phosphotungstate [H_7_P_8_W_48_O_184_]^33−^ (abbreviated as P_8_W_48_), and Co(II)‐containing silicotungstates, on GCEs for subsequent electrocatalytic water reduction application in acidic solution.^[^ ^7c^ ^]^ On the basis of electrochemical investigation the observed activity was attributed to POM's proton and electron reservoir‐like properties.^[^ ^127^ ^]^ Recently, a hybrid of P_8_W_48_ and reduced graphene oxide sheets (rGO) (**Figure** **7**a) has been reported as an efficient electrocatalyst for improved HER in an acidic medium. In this report, POM was immobilized in 3D configuration on rGO to put forward an experimental confirmation of “‘microenvironment effect”’ and to highlight its significance in the development of robust electrocatalysts.^[^ ^123^ ^]^ Electron‐storage or accepting feature of graphene together with its large surface area presents graphene as an ideal material to design electrochemical materials.^[^ ^128^ ^]^ Surface oxygen functionalities of graphene oxide facilitate immobilization of different electroactive catalysts but the availability of a high degree of oxidation considerably disrupts the electronic p‐delocalization. Reduced graphene oxide shows partial disruption in its charge‐flow, which in turn affects electrochemical properties.^[^ ^129^ ^]^ In 2018, Fernandes and his team reported their work on polyoxometalate‐graphene Electrocatalysts for HER in acidic medium.^[^ ^130^ ^]^ In this work, three different POMs were immobilized on oxidized graphene flakes to explore shape/size/composition/charge–activity relationships. Recently, a one‐pot method has been developed to synthesize an Au@Pd/PMo_12_/rGO nanocomposite catalyst. In this work, Keggin‐type phosphomolybdic acid (PMo_12_) was used to accomplish both the reduction as well as stabilization of the prepared composite. Electrochemical studies revealed remarkable activity of nanocomposite, Au@Pd/PMo_12_/rGO, for HER over a wide pH range and this high efficiency together with stability suggested an output of a synergistic effect emerged from the core@shell structure of the Au@Pd on rGO, achieved with the redox active nature of PMo_12_. In addition, composite catalysts presented low overpotentials −109, 250, and 300 mV versus RHE in acidic, neutral, and basic media, respectively, for a current density of 10 mA cm^−2^. Also, the ability of the electrocatalyst to provide high HER current density and its remarkable stability has been confirmed.^[^ ^131^ ^]^ Heteropolyanions (HPA) and carbon nanotubes (CNTs) have become very attractive in many fields.^[^ ^132^ ^]^ The high surface area, appreciable electronic conductivity, and superior mechanical properties of CNTs introduce them as excellent candidates to accelerate electron transfer reactions.^[^ ^133^ ^]^ POM‐based compounds have been explored over the years for metallic^[^ ^134^ ^]^ and semiconducting electrodes.^[^ ^132^, ^135^ ^]^

Redox tuneability and diverse coordination chemistry of POMs can originate multifunctional materials after coordinating with different organic linkers to achieve pre‐judicious properties.^[^ ^136^ ^]^ POM‐based MOFs (POMOFs) combine the redox nature of the POM units and high surface area together with the permanent porosity of a MOF, which may favor hydrogen generation as an electrocatalyst.^[^ ^51^ ^]^ *ε*‐Keggin POMs, [*ε*‐PMo^V^_8_Mo^VI^_4_O_40‐x_(OH)_x_M_4_] (M = Zn^II^,La^III^), comprises of a core of an *ε*‐Keggin unit that is capped with four metal ions (M) and therefore such ε‐Keggin ions can be used as anionic (M = Zn, x = 0) or as cationic (M = Zn, La, x = 3‐5) building blocks.^[^ ^137^ ^]^ Dolbecq and his coworkers have reported three POMOFs for HER in acidic.^[^ ^138^ ^]^ In this work, triangular 1,3,5‐benzene tricarboxylate linkers (denoted as trim) were grafted on tetrahedral Zn‐ε‐ POMs under hydrothermal conditions.

(TBA)_3_[PMo^V^_8_Mo^VI^_4_O_36_(OH)_4_Zn_4_][C_6_H_3_(COO)_3_]_4/3_.6H_2_O (*ε*(trim)_4/3_) revealed a 3D open‐framework comprised of molecular Keggin fragments connected with organic linkers (1,3,5‐benzene tricarboxylate = trim), with tetrabutylammonium counterions (TBA^+^) residing inside the open channels. ε(trim)4/3 is a novel (3,4)‐connected net, named ofp for open‐framework polyoxometalate, where POM's trapping in 3D configuration, known as microenvironment effect, was observed as a responsible factor for their stability and enhanced electrocatalytic activity (Figure 7b). A remarkable electrocatalytic efficiency for HER was observed in acidic electrolytes.^[^ ^138^ ^]^ Qin and his team members reported two novel POMOFs, [TBA]_3_[*ε*‐PMo^V^_8_Mo^VI^O_36_(OH)_4_Zn_4_][BTB]_4/3_•*x*Guest (NENU‐500, BTB = benzene tribenzoate, TBA^+^ = tetrabutylammonium ion) and [TBA]_3_[*ε*‐PMo^V^_8_Mo^VI^O_37_(OH)_3_Zn_4_][BPT] (NENU‐501, BPT = [1,1′‐biphenyl]−3,4′,5‐tricarboxylate), where POM units served as nodes to direct connect the organic ligands to maintain 3D open frameworks, and TBA^+^ ions, exited inside balance the anionic frameworks, not only maintained stability in air but also gifted tolerance in acidic and basic media. Remarkably, NENU‐500, a three‐dimensional hydrogen‐evolving cathode, demanded only an overpotential (*η*) of 237 mV to attain 10 mA cm^−2^ in acidic electrolyte for HER. Both NENU‐500 and NENU‐501 showed no change in their activities even after the 2000 cycles in acidic electrolyte.^[^ ^51^ ^]^ Moreover, well‐known layer‐by‐layer technique has been applied to alternatively adsorb the POM, [(TBA)Mo_12_(AleThio)_4_], and a polymer (polyethyleneimine = PEI) (Figure 7c). The as obtained nanolayered electrode revealed higher electrocatalytic HER activity (−0.077 V at 10 mA cm^−2^, and a low Tafel slope of 80 mV dec^−1^) in acidic electrolyte, which was attributed to the synergetic effect of the positively charged PEI polymer and the catalytically active Mo‐POM.^[^ ^59^ ^]^

H_2_ production without oxygen impurity is a grand challenge.^[^ ^139^ ^]^ Natural photosynthetic systems have ability to separately produce O_2_ and H_2_ gases via water splitting.^[^ ^140^ ^]^ An approach of electron‐coupled‐proton buffer (ECPB) can facilitate the separate production of hydrogen and oxygen gases via water electrolysis at different times.^[^ ^139^ ^]^ [PMo_12_O_40_]^3−^ has been explored as ECPB for water splitting to produce separately H_2_ and O_2_ gases at completely different times. In this approach, electrons and protons, obtained during the water oxidation to generate oxygen gas, were taken up reversibly by ECPB, rather than being consumed directly to generate H_2_. After that ECPB was re‐oxidized to release these protons and electrons to generate H_2_ gas.^[^ ^139^ ^]^ Similarly, a Keggin‐type Zinc‐based POM (H_6_ZnW_12_O_40_) has been reported to improve the ECPB‐mediated water‐splitting process.^[^ ^54^ ^]^ This work highlights the role of heteroatoms in tuning the redox features of POM architectures, which in turn doubles, from two to four electrons, the numbers of electrons and protonation in redox processes.^[^ ^141^ ^]^ Successful tuning of redox features of POMs has been achieved with the substitution of P^5+^, Si^4+^, and Zn^2+^ as heteroatoms, where Zn‐POM (H_6_ZnW_12_O_40_) has revealed high decoupling efficiency and electrochemical energy efficiency as 95.5% and 83.3%, respectively, in ECPB‐mediated water splitting (Figure 7d). This work demonstrates the significance of heteroatoms in tuning the redox properties of POM architectures and opens new corridors to generate hydrogen gas from safety and efficiency viewpoints.^[^ ^54^ ^]^ The composites catalysts NiM‐POM/Ni foam (M = Co, Zn, Mn) have been reported to reveal excellent durability and activity in 1.0 m KOH, where NiCo‐POM/Ni foam composite (Figure 7e) outperformed (an overpotential of 64 mV at 10 mA cm^−2^ versus RHE) among all catalyst samples.^[^ ^53^ ^]^ In the electrocatalyst designs, POM behaves as a stabilizing agent during the water reduction process.^[^ ^142^ ^]^ Redox‐active molecular POMs have the ability to enhance proton transfers and further benefit the H atom absorption, hence firm linkages of POMs to Cu or Co‐containing species can provide required H‐atom absorption sites together with multiple electron transfers to achieve lower overpotential for HER (Figure 7f‐g).^[^ ^56^, ^143^ ^]^ An Anderson‐type POM, NiMo_6_O_24_, based electrocatalyst (NiMo_6_O_24_@Cu/TNA) obtained on the fabrication of POM modified‐Cu dendrites on TiO_2_ nanotube array (TNA) (Figure 7g).^[^ ^144^ ^]^ Under an acidic environment, POMs (NiMo_6_O_24_) perform as Bronsted acid to facilitate multiple H^+^ transfers and thus assist the H atom absorption, which eliminates the issue of the H atom absorption capacity of Cu‐species. In response to a negative potential scan, POM reduces at first and then transfers electrons to accelerate H atom absorption, subsequent H_2_ generation release lowers the overpotential of HER.^[^ ^144^ ^]^ Redox tunability of POMs is highly cherished in designing robust electrocatalytic materials.^[^ ^130^ ^]^ Hence, designing inexpensive electrocatalysts for HER is still a grand challenge due to insufficient activity and low stability at high proton concentrations.^[^ ^7b^ ^]^ Recently, an electroactive sandwich‐type POM (Na_10_[Mn_4_(H_2_O)_2_(VW_9_O_34_].26H_2_O) was anchored on the CoSe_2_‐nanobelts to achieve stable and efficient HER active electrocatalyst (Figure 7f). The high durability was attributed to the strong coordination chemistry of POMs, and the improved HER efficiency was considered an outcome of the synergistic effect emerged from the stabilized POMs that facilitated PCETs prior to the molecular hydrogen evolution.^[^ ^56^ ^]^

Among the noble‐metal based POMs, polyoxopalladates(II) comprise the largest number of members. The parent palladate POM, 13‐palladate [Pd^II^_13_As^V^_8_O_34_(OH)_6_]^8‐^ or (Pd_13_As_8_), comprise of a central Pd^II^ guest, a cage of cub octahedral [Pd_12_] and eight capping groups AsO_4_, to design a cubic shape of POM architecture.^[^ ^146^ ^]^ The investigations have revealed that capping groups, [AsVO_4_]^3−^, can be replaced with [Se^IV^O_3_]^2−^ or [PhAs^V^O_3_]^2−^ units^[^ ^147^ ^]^ 15‐palladate [Pd_15_P_10_O_50_]^20−^ (“Pd_15_P_10_”) was designed from (“Pd_13_As_8_”) with replacement of arsenate heterogroups by phosphate ions.^[^ ^147^ ^]^ Similarly, [Pd_15_Se_10_O_40_Na]^9−^ architecture was developed from Pd_15_P_10_ .^[^ ^148^ ^]^ Furthermore, central palladium ion in [Pd_13_(PhAs)_8_O_32_]^6−^ framework can be substituted with lanthanide and other transition metal ions, and hence a general formulation for such architecture is MPd_12_L_8_ (M = central metal ion, L = capping groups).^[^ ^149^ ^]^ Afterwards, a Cu(II)‐containing polyoxo‐22‐palladate(II), [Cu^II^_2_Pd^II^_22_P^V^_12_O_60_(OH)_8_]^20−^ = (Pd_22_Cu_2_P_12_) was prepared using one‐pot method (Figure 7h). In this case, POM was anchored in a carbon paste electrode (CPE) for HER application at pH = 7.1. In this work, cubic [Pd_12_L_8_] shell was explored to incorporate different guest cations (M) and hetero‐groups (L), in order to develop novel double‐cuboid‐shaped Pd_22_Cu_2_P_12_. This novel POM incorporated the largest number of palladium ions yet found in palladium‐based POMs.^[^ ^145^ ^]^

### 4.7 POM‐Based HER Electrocatalysts versus State‐of‐the‐Art Counterparts

Although the OER is considered as an efficiency‐limiting reaction in electrolytic water splitting, the HER, which also involves several steps, poses many challenges that must be addressed. Presently, Pt/C is used as a commercial catalyst for HER, however, low reserves and the high cost of Pt strictly hamper its large‐scale industrial application.^[^ ^150^ ^]^ Currently, in industry, alkaline electrolytes are used for electrocatalytic HER owing to the issues of corrosion of equipment and acid mist contamination in the acidic electrolytes.^[^ ^151^ ^]^ However, in alkaline media, the catalytic activity of Pt is hampered by the water dissociation because of the lack of oxyphilic surfaces to break the O−H bond of H−O−H.^[^ ^152^ ^]^ It has been found that electrode/electrolyte interface potential induces a strong thermodynamic force which subsequently causes the dissolution of the electrocatalysts. Therefore, new advances are suggested to prohibit the dissolution of electrocatalysts and consequently activity loss. The excellent electron‐sponge feature of POMs makes them perfect for reversible multielectron transfer, owing to the incorporation of highly redox‐active metals in POM's 3D structural framework, hence fulfills the requirement of PCET, and giving rise to technologically applicable electrocatalysts for the water splitting as well as for oxidation and reduction reactions in fuel cells.^[^ ^123^ ^]^ POMs are potential candidates as electrocatalysts for the cleanest H_2_ production and have close enough performance to the most used commercial electrocatalyst (Pt/C), while being considerably cost‐effective. The studies discussed in the section “POM‐Based HER electrocatalysts” demonstrate the excellent potential of POMs for electrocatalytic HER, and encourage further advancements toward POM‐based nanocomposites. The HER performances of POM‐based catalysts especially at high‐current densities have gained a significant boost. The inorganic constituents POM's framework (Figures 4 and 7) hinders the structural degradation during the electron/release process, therefore POMs‐POM‐based materials have been widely used as durable HER catalysts. Ideally, anchoring POMs on conductive substrates of larger surface area has resulted in the development of new and advanced POM‐based HER electrocatalysts which, unlike traditional catalysts, have revealed good performance even at high proton concentrations, where most of the catalysts suffer deterioration and present insufficient activity.^[^ ^7^, ^56^ ^]^

### 4.8 Theoretical Studies of POM‐Based OER and HER Electrocatalysts

**Figure 8:**
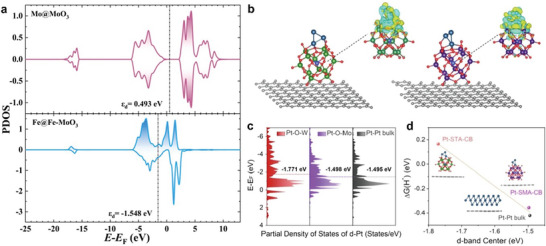
DFT studies of POM‐based OER and HER electrocatalysts. a) PDOS of Mo atom in MoO_3_ and Fe atom in Fe@MoO_3_. Reproduced with permission.^[^ ^152^ ^]^ Copyright 2021, Elsevier. b) The difference in charge density between Pt_3_ cluster and the STA/SMA‐CB, and the configurations of Pt‐STA‐CB and Pt‐SMA‐CB electrocatalyst systems. Gray, red, blue, green, purple, and dark cyan balls represent the C, O, Si, W, Mo, and Pt atoms, respectively. c) PDOS plots and *ε*_d_ of Pt atoms on Pt‐STA‐CB, Pt‐SMA‐CB, and Pt/C. (d) A scaling relationship between *ε*_d_ and the ΔG_H*_. Reproduced with permission.^[^ ^153^ ^]^ Copyright 2022, Elsevier.

To gain a deeper understanding of the electrocatalytic OER/HER performances on the electrocatalytic materials of POMs, DFT calculations are performed to assess the adsorption strength of the reaction intermediates.^[^ ^33a^ ^]^ According to the d‐band center (*ε*_d_) theory, the lower *ε*_d_ level reveals the weak adsorption of (reaction) intermediate species that is desired for high catalytic performance. In order to investigate the electrocatalytic activity on a theoretical basis, the partial density of states (PDOS) of active (metal) sites are calculated and analyzed, where smaller values of *ε*_d_ predict weak adsorption of intermediates species, thus subsequently easy desorption of the adsorbed species.^[^ ^153^ ^]^ In addition, bandgap calculations are performed to estimate the electron transport behavior and thus enhance the conductivity of the catalytic materials. The catalyst with smaller bandgaps reveals accelerated electron transport and thus improved conductivity, which is imperious for the electrocatalysis process.^[^ ^153^ ^]^ To assess the OER electrocatalytic activity of an Anderson‐type polyoxometalate‐derived catalyst, the PDOS of active metals (Fe/Mo) have been reported.^[^ ^153^ ^]^ In this report, *ε*_d_ calculation revealed that Fe (*ε*_d_ = −1.548 eV) has a smaller value of *ε*_d_ than the Mo (*ε*_d_ = 0.493 eV) (**Figure** **8**a). Moreover, DFT calculations revealed that Fe@MoO_3_ has a smaller bandgap of 0.70 eV than that of pristine MoO_3_ (2.77 eV). It can be concluded that integration of Fe with MoO_3_ species via O–FeO– bridge significantly promotes the 1) desorption of the adsorbed species, 2) electrons transport capabilities of the catalyst, hence 3) boosting the electrical conductivity of the electrode, which collectively results in the enhancement of OER activities.

Recently, atomic cluster‐doped Keggin POMs were reported for electrocatalytic HER.^[^ ^154^ ^]^ In this work, Pt atomic clusters were anchored on the polyoxometalates‐carbon black (Pt‐POMs‐CB). DFT studies show that the Pt‐clusters were stabilized on the surface due to charge transfer from Pt to O atoms of the POMs. For DFT calculations two W and Mo‐based Keggin POMs, namely silicotungstic acid (STA) and silicomolybdic acid (SMA), were used. The calculation of adsorption energies of Pt_3_‐cluster on STA‐carbon black (STA‐CB) (−9.81 eV) and SMA‐carbon black (SMA‐CB) (−10.21 eV) indicated that the interaction of Pt_3_‐cluster with STA/SMA is much stronger than the cohesive energy of bulk Pt‐atoms. Figure 8b shows the charge density difference between Pt_3_‐cluster and the STA/SMA‐CB in the STA/SMA‐CB catalyst. Then, we calculated the adsorption free energy of H* (Δ*G*_H*_), the *ε*_d_ of the metal (Pt) sites, and the reaction mechanisms for HER. The *ε*_d_ was calculated and the PDOS of Pt *d*‐orbitals for Pt‐STA‐CB, Pt‐SMA‐CB, and Pt/C were plotted to exhibit the adsorption of H* (Figure 8c), where Pt‐STA‐CB shows a downshift of *ε*_d_, which was attributed to the charge transfer from Pt to O atoms of POM framework. Importantly, it was found that compared with Mo atom, W atom transfers less charge to O atom, thus O atoms of STA get more charge from the Pt atoms and cause a downshift *ε*_d_ of Pt in the Pt‐STA‐CB system. It is known that the downshift of *ε*_d_ weakens the adsorption strength reaction intermediates.^[^ ^153^ ^]^ Likewise, the *ε*_d_ downshift in Pt‐STA‐CB system weakened the adsorption strength of H* and hence improved the electrocatalytic HER performance. A scaling relationship between the *ε*_d_ (eV) and the adsorption‐free energy of H* (Δ*G*_H_) on Pt atoms (Figure 8d) is also showing a good consistency with the above findings.^[^ ^154^ ^]^

## 5 Information Storage

The variety of data types and exponential growth of data sizes have originated alarming challenges in data storage techniques. To address such challenges, scaling down is generally considered as most viable approach which offers integration of multiple memory cells per unit area.^[^ ^155^ ^]^ But this approach is, based on Moore's Law (1965) which states that the number of transistors on a single chip of silicon will double every two years and hence, the chip's computing performance will double too, nearing to its end owing to associated technical complexities, including physical squeezing limitations and thermodynamic effects, as it is warned by “International Technology Roadmap for Semiconductors”.^[^ ^12a,c^ ^]^ Importantly, the key issues related to the decrease of cell dimensions such as intense leakage current and device‐to‐device discrepancies owing to non‐uniform Si‐doping, need to be eradicated by integrating appropriate molecules, comprising superior electronic architectures and non‐size dependent features, in next‐generation memory devices. An urgent and alternative route to tackle this grand challenge is to design molecular memories with highly dense data storage abilities. Molecular memories have the capabilities to perform with few‐electrons at the molecular levels and therefore, offer ultra‐dense system compatibilities with low power consumption.^[^ ^156^ ^]^ Designing molecular electronic devices involves a systematic understanding of electrical features of materials from atom to macroscopic levels,^[^ ^157^ ^]^ accurate handling of the architectural and interfacial morphologies,^[^ ^158^ ^]^ and consideration of stability and reduction of cell‐cell variability.^[^ ^159^ ^]^ The concept of molecular electronics appeared for the first time in 1973, when Aviram and Ratner constructed an electronic device based on a single molecule.^[^ ^46^ ^]^ Ever since the discovery and development of molecular electronics, the developing area of molecular electronics employs switchable molecules as data storage elements between nano‐electrodes to exhibit molecular‐based random access memory (RAM).^[^ ^160^ ^]^ Numerous efforts have been devoted to enhance memory windows, multibit data storage capability, flexibility, and acquire the performance of molecular memories developed of small molecules^[^ ^161^ ^]^, conjugated materials,^[^ ^162^ ^]^ dimensional materials,^[^ ^163^ ^]^ and electrets with versatile architectures, including sandwiched resistive switching memories and floating gate memories.^[^ ^18^, ^164^ ^]^

Memory devices are categorized on the basis of two criteria: i) whether it is a charge‐storage type or resistance‐chancing type and ii) whether it is volatile or nonvolatile.^[^ ^165^ ^]^ Noticeably, in volatile memory (VM), all data stored is lost when the device is powered off (i.e., static RRAM (SRAM)) unless the devices are periodically refreshed (i.e., dynamic RAM (DRAM)), while in non‐volatile memory (NVM). NVMs (i.e., Flash memories) the information stored is retained even after the power supply is turned off.^[^ ^166^ ^]^ The applications of charge‐based memory devices, such as DRAM and flash memory, in large‐scale integration technology may soon be restricted owing to their bigger size and higher power consumption. In order to obtain well‐suited memory devices for future high‐frequency nanoelectronics, numerous unconventional devices including ferroelectric RAM (FRAM), magnetoresistive RAM (MRAM), phase‐change RAM (PRAM), and resistive random‐access memory (RRAM), have been proposed for storing digital information.^[^ ^167^ ^]^ Among all aforementioned NVMs, RRAM has exceptional capability to exhibit high processing‐speed (sub‐ns), high endurance (> 1012 cycles), low energy consumption (< 0.1 pJ). Therefore, RRAM is considered as one of the most imperious candidates for next‐generation NVMs.^[^ ^168^ ^]^ RRAM, a type of NVM, involves internal redox reactions to induce resistance switching phenomenon, which relies on the interplay of electrochemical and thermochemical effects.^[^ ^169^ ^]^ In general, an RRAM cell comprises a conductor/insulator (or semiconductor)/conductor sandwich structure. The intrinsic physical phenomenon behind RRAMs is resistive switching (RS), which means that the device can be freely programmed into HRS (or OFF state) or LRS (or ON state) under the influence of external electrical stimuli. In most cases, the current flows uniformly through the device in the HRS and is restricted to a local region with high conductance known as a conductive filament (CF) in the LRS.^[^ ^168^ ^]^

### 5.1 Nonvolatile Neuromorphic Memory

The ability of human brain to perform complex processing with less energy consumption (5 orders of magnitude) than the supercomputers, has urged scientists to develop new nanoelectronics that could imitate the performance of the human brain, which has led to neuromorphic computing. Valuably, the low‐power consuming neuron‐spike‐based computation of the human brain delivers superior energy efficiency.

To mimic the working of neurons, which serve as integrator and spiking devices in the biological system, a memristor (memory + resistor),^[^ ^170^ ^]^ an electrical device component that has a capacitor‐like architecture in which an active layer is sandwiched between two conductive electrodes, is implemented in a neuromorphic system.^[^ ^171^ ^]^ In the biological brain, synapses provide dynamic interconnections between the neurons. However, in neuromorphic systems, a memristor substitutes the functioning of a biological synapse. Memristor exhibits a resistance (synaptic weight) that changes in response to an applied periodic voltage (or current), and the resultant state of resistance is maintained even when the power is turned off.^[^ ^20^ ^]^ The resistance of RRAM, a memristor with small footprints and lower power consumption without compromising other metrics,^[^ ^172^ ^]^ switches between HRS and LRS when a positive or negative voltage is applied, and creates a conductive pathway, known as CF.^[^ ^44^ ^]^ Nonvolatile neuromorphic devices incorporate redox‐active metal oxide materials to achieve HRS and LRS switching under the influence of applied voltage, and thus work as a substitution to a biological synapse.^[^ ^20^ ^]^

One of the substantial advantages of the RRAM principle is that it allows scalability down to the nanometer level,^[^ ^173^ ^]^ which permits the integration of molecular clusters, POMs, as resistive switching centers. POMs as soluble molecular semiconducting oxides, possess a 3D framework with discrete and stable redox states that can be tuned via appropriate dopants, thus satisfying prerequisites to overcome the challenges of future's molecular electronics. Remarkably, the discrete spin states of POMs and their derivatives make them appealing candidates for applications in next‐generation neuromorphic computing.^[^ ^174^ ^]^

### 5.2 Information Storage at Molecular‐level

The smart tactic of miniaturization of floating gate devices to achieve improved performance is near to its end owing to inevitable technical issues. Designing smaller and more efficient devices with the hybrid silicon/molecular approach is the most promising strategy to replace silicon floating gates with redox‐active molecules.^[^ ^175^ ^]^ Incorporation of redox‐active molecules into silicon structures creates a new class of molecular electronic devices, where redox‐active molecules act as molecular‐scale information storage systems.^[^ ^175^, ^176^ ^]^ Generally, hybrid complementary metal oxide semiconductor (CMOS)/molecular memories involve a self‐assembled monolayer of memory elements, the redox‐active molecules, to exhibit several charge storage states in a narrow potential range. In response to an oxidizing voltage, the monolayer of redox‐active molecules loses electrons and turns to a positively charged monolayer, while the application of a reducing voltage causes the electrons to transfer to molecules in order to attain a neutral state. These two states of absence and presence of electrons, in metal‐oxide monolayer, are represented by “1” and “0” respectively, into a conventional floating gate.^[^ ^177^ ^]^ The basic paradigm for storing electronic information to a molecular‐scale memory is the retention of charge at the molecular level. This approach involves multiple oxidation states of individual redox‐active molecules to store charge, where several oxidation states within one molecule can be utilized to access >1 bit.^[^ ^178^ ^]^

The RS process is crucial for next‐generation nanoelectronics, for example, information storage, anticounterfeiting, and molecular switching devices. However, the RS process in most of the commercial memory devices remains active below 125 °C, and only a few devices retain RS behavior at high temperatures (≈200 °C).^[^ ^179^ ^]^ Recently, numerous advances have been made but most of the attention remained on improving the RS performance at normal temperature, and the stabilities under harsh environments such as high temperature have received much less interest.^[^ ^180^ ^]^ The precise designing of advanced nanoelectronics to perform under harsh conditions, requires the integration of redox‐active species between the layers of optically, electrically, and thermally stable active species, to serve as both deep charge traps and carriers under external voltage.^[^ ^181^ ^]^ The ability of molecular POMs to establish coordination and, subsequently, multiple charge transfers between the active layers, has opened the opportunities for 3D POMs to develop nonvolatile neuromorphic devices for high‐density information storage.^[^ ^179^ ^]^

### 5.3 Mandatory Features of a Material for Nonvolatile Neuromorphic Devices

Generally, a material with a high surface‐to‐bulk ratio, multiple redox states, and compatibility with

traditional CMOS technologies is considered a promising candidate to develop neuromorphic

devices. The mandatory features of a material for neuromorphic devices are given below;

1. Working of RRAMs is based on multiple switching of HRS to LRS and vice versa; each switching process causes a small permanent damage to the RRAMs, which ultimately lowers the device efficiency, thus, the maximum number of switching cycles until failure is known as endurance.^[^ ^182^ ^]^ The RS behavior depends on the ability of a material to undergo multielectron uptake and release without structural deterioration.^[^ ^35^ ^]^
2. Data retention of RRAMs is defined as the period over which the HRS and LRS of the memory device remain stable after undergoing SET and RESET switching, irrespective the device is electrically powered ON or OFF. Retention time shows the intrinsic ability of a memory cell to retain its content.^[^ ^183^ ^]^ It is essential for a material to maintain its programmed state over an extended period.
3. Multilevel storage reveals the capability of a memory cell to achieve reproducible resistive switching between multiple resistance states and, subsequently, store multiple values. Multilevel storage increases the storage density, but needs a large enough resistance‐ratio between each state to enable an external circuit to discriminate between states.^[^ ^183^ ^]^ The ability of a material to accept multiple electrons and maintain their delocalization is essential to obtain multibit data storage.^[^ ^35^ ^]^
4. A switching time between two states, HRS and LRS, defines the efficiency of the RRAM devices.^[^ ^184^ ^]^ Among various types of RRAMs, the conductive filament type RRAMs are capable of exhibiting the switching speed in picoseconds, hence making them one of the fastest and most efficient RAMs.^[^ ^185^ ^]^ Fast switching speeds for a material to enable quick read and write operations is highly desirable to achieve high efficiency of the memory device.
5. The large‐scale applications of memory devices depend on the power consumption during a switching between SET and RESET process. Among various classes of memory devices, the power consumption of RRAMs is usually very small. To measure the power consumption in RRAMs, a compliance current (Icc in µA) is applied, so that, after setting the device to LRS, the current will not be so large to cause irreversible device breakdown. The low power consumption (µW), during the setting process (Pset (µW) = V_set_ × I_cc_) and the resetting process (Preset (µW) = V_reset_ × I_reset_), suggests the suitability of the memory device for commercialization.^[^ ^167^ ^]^ Highly redox‐active metal oxide materials such as POMs have the ability to facilitate low‐power programming/erase cycles to decrease energy consumption.^[^ ^35^ ^]^
6. One of the main reasons that hinders the applications of RRAMs is the poor inhomogeneity during the fabrication process, leading to a high degree of variability in switching voltage.^[^ ^186^ ^]^
7. The RS process is crucial for NVM devices. A material should have high thermal stability. Typically, a contender materials must be capable to perform multielectron uptake and release at high temperatures to facilitate RS from room temperature to high temperatures above 150 °C.^[^ ^179^ ^]^
8. Smaller cell size requires less space on the Si wafer, thus improving the device yield at a low cost. Moreover, decreasing the size of metal‐ion‐based filament to the atomic level gives a possibility to form an electrically controllable break junction, to detect spin‐like switching behavior in RRAM.^[^ ^187^ ^]^ As memory devices continue to evolve towards higher densities, the properties of a candidate material need to be stable and consistent even at nano/sub‐nano scale, to realize their precise integration into designs of advanced memory devices.

### 5.4 Traditional Materials for NVM Devices

#### 5.4.1 Organic–Inorganic Hybrids

The advantages of organic materials including high flexibility, low cost, and easy solution processability, confirm their prospect towards next‐generation memory devices. Metallophthalocyanines, 2D planar semiconducting molecules, are well‐known for self‐assembly stacking owing to their capability of π–π interactions.^[^ ^188^ ^]^ The excellent thermal and chemical durability, as well as unique optical and electrical features of MPc and hyperbranched MPc polymer thin‐films enable them to exhibit electricity‐induced metastable resistance states for data storage.^[^ ^189^ ^]^ However, limited switching speed, poor reproducibility, insufficient write–erase stability, or the need of high energy and area, hinder the overall commercialization of organic molecule‐based memory devices.^[^ ^190^ ^]^ Over the years, organic–inorganic hybrid perovskites have gained increasing attention toward RRAM, Artificial Synapse, and Logic Operation.^[^ ^191^ ^]^ Among various organic–inorganic hybrid materials, CH_3_NH_3_PbX_3_ (X: I^−^, Br^−^ or Cl^−^) based hybrid perovskites have been explored more owing to their attractive feature of bandgap tunability, long charge diffusion length, magnetic, and dielectric polarization which are associated with the CH_3_NH_3_^+^ group.^[^ ^192^ ^]^ However, the perovskite oxide‐based RRAM devices are imperfect due to many associated disadvantages, such as the need of high temperature for processing, and rigid ceramic feature of the switching layer.^[^ ^193^ ^]^ Many efforts have been directed to explore outstanding properties, for instance, long‐range charge diffusion length, high carrier mobility, and etc., of hybrid perovskite‐type materials.^[^ ^194^ ^]^ However, from a design perspective, overreliance on organic molecular electronics has encountered serious limitations associated with the organic molecules such as high resistance, low efficiency, poor endurance, prolonged switching times, unsatisfactory stability, and difficult fabrication together with reproducibility and reliability related issues.^[^ ^168^, ^195^ ^]^

#### 5.4.2 Traditional Metal Oxides

Since 1962, when Hickmott investigated resistive switching behavior^[^ ^19b^ ^]^ in binary oxides, the phenomenon of resistive switching has been examined in several binary metal oxides such as ZnO, NiO, MgO, CuO, HfO_2_, Al_2_O_3_, TiO_2_, Ta_2_O_5_, Nb_2_O_5_, Fe_2_O_3_, and many more. Among all ZnO, a wide‐bandgap material, gets an edge for the same owing to its fabrication‐friendly features.^[^ ^196^ ^]^ Therefore, scientists have largely studied ZnO with a variety of expensive electrodes such as Ag, Au, and Pt, and inexpensive electrodes for RRAM applications.^[^ ^197^ ^]^ Despite numerous advances, exploring novel materials that could break through the limitations of oxide‐based memristors, such as low reproducibility and high‐forming voltage, is highly imperious. Among most of the novel materials, such as perovskites, 2D materials, biomaterials, and so on, which have been explored for RRAMs applications,^[^ ^198^ ^]^ POMs are considered as superior contender materials to design next‐generation data storage devices.

### 5.5 Characteristic features of POMs for Nonvolatile Neuromorphic Devices

Thermal stability, structural diversity, and redox tune‐ability of POMs have attracted interest from different research domains owing to remarkably unmatched physical and chemical properties, and therefore have been considered as prime candidates in a variety of fields of research.^[^ ^39^, ^199^ ^]^ Molecular POMs, comprise distinct nano‐sized architectures with unique and attractive electronic features, have inherited capabilities to satisfy the requisites in the development of integrated nanosystems.^[^ ^63^, ^200^ ^]^ POM chemistry is a key research area that has unrivaled potential to develop sophisticated molecular materials and devices, therefore, has earned popularity in the fields of nanoscale science and electronics.^[^ ^195c^ ^]^ The electrochemical, electrochromic, optical, photochromic, and magnetic properties of POMs have been largely explored, and thereby, POMs have been widely accepted to be essential constituents of next‐generation electronics.^[^ ^27^, ^201^ ^]^ As electroactive and redox‐active molecules, POMs are generally featured by reduction processes and are regarded as zero‐dimensional n‐type semiconducting materials which exhibit very low charging energy, generally assessed as the energy gap between the *E*_F_ of metal electrode and the lowest unoccupied molecular orbital (LUMO) level of POMs.^[^ ^50^, ^202^ ^]^ POMs demonstrate diverse characteristics, highly appreciable for applications in the field of electronics.

1. Nanosized, ≈1 nm, well‐defined molecular frameworks.^[^ ^202b^ ^]^
2. POMs unlike the classical small inorganic anions such as NO^3–^, SO_4_^2–^, or PO_4_^3–^ exhibit totally different electronic properties owing to their low charge density supported by the available oxygen atoms in the POM architecture. Attractively, the existence of low‐lying orbitals at the metal ions of POM architecture tolerates multiple electron reductions without bringing any structural changes, and therefore, POMs offer uptake and release on many electrons, endorses POMs electron sponge‐like nature with electron mobility as high as 4 × 10^−3^ cm^−2^ V^−1^ s^−1^ a, suggesting their applications in electronic devices.^[^ ^32^, ^50^, ^200^, ^203^ ^]^
3. After reduction processes, the accepted electrons can be delocalized over the metal centers of POM architecture, a vital feature that is highly demanding for charge confinement applications. POMs behave as charge detention units in flash memories, and thus POMs integration offers long‐term stability upon programming/erasing cycles.^[^ ^204^ ^]^
4. Thus, ability to act as the charge trapping modules that assist the formation of CFs, due to their delocalized electrostatic adsorption property.^[^ ^58^ ^]^ The formation of CFs is essential for resistive switching (RS) of a memory device, which means that the device can be freely programmed into a high resistance state (HRS, or OFF state) or a low resistance state (LRS, or ON state).^[^ ^168^ ^]^
5. Effectively accessible multiple redox states, a characteristic for application in high‐density data storage.^[^ ^50^ ^]^
6. Exceptionally configurable molecule with ease of practical integration in CMOS technology.^[^ ^205^ ^]^

Chemistry of inherently high number of oxygen atoms in cooperation with transition metal “addenda” atoms of POM frameworks reveals excellent electronic complementarities with SiO_2_ and facilitates POMs integration with the stacking materials through covalent or electrostatic interactions,^[^ ^206^ ^]^ spin‐coating,^[^ ^34^ ^]^ drop‐casting,^[^ ^205^ ^]^ and layer by layer self‐assembly.^[^ ^207^ ^]^ Scientific investigations have revealed that the incorporation of molecular POMs in the metal oxide–semiconductor (MOS) configuration has the ability to eradicate the challenges associated with the organic molecules, power consumption as well as performance‐related issues of electronic devices.^[^ ^208^ ^]^

### 5.6 POM‐Based NVM Devices

Architectural diversity and versatile redox‐active features of POMs permit firm linkages with silicon platforms. Unlike organic molecules, redox‐active molecular POMs require no organic‐linker and high temperatures to establish coordination with silicon structures.^[^ ^176^, ^209^ ^]^ Hence, the integration of redox‐active molecular POMs to CMOS platform is an attractive approach to design next‐generation memories. From development perspective, the position of LUMO level of POMs is very attractive in designing molecular memory devices. Generally, the required external‐bias to induce an irreversible reduction of molecular POMs is largely influenced with the electron injection/extraction barrier, a gap between two energy levels.

**Figure 9:**
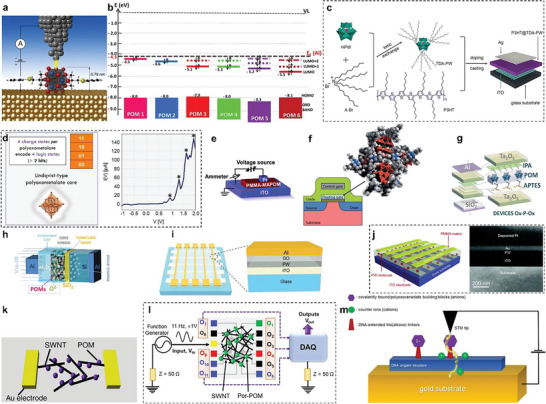
POM‐based NVMs. a) Single‐molecule of a Vanadium‐POM sandwiched between the Au (bottom) and Pt (top) electrodes (where Pt belongs to tip of scanning tunneling microscope (STM). Reproduced with permission.^[^ ^210^ ^]^ Copyright 2018, American Chemical Society. b) Molecular orbital diagrams of POMs, showing HOMO and LUMO levels. Reproduced with permission.^[^ ^74^ ^]^ Copyright 2015, American Chemical Society. c) Preparation of P3HT@TDA‐PW nanocomposite device. Reproduced with permission.^[^ ^57^ ^]^ Copyright 2022, John Wiley and Sons. d) The Lindqvist‐type POV6 with a diamagnetic V_6_O_19_ core, stores 2 bits of information for its 4 electrically generated logic states at room temperature and < 2 V. While I–V graph (right side of Figure 9d), shows 4 conduction states (*) correspond to single electron transfers from the STM tip into empty molecular orbitals of vanadium atoms of POV6. Reproduced with permission.^[^ ^73^ ^]^ Copyright 2023, IEEE. e) Design of a PMMA–MAPOM composite sandwiched between Pt and ITO electrodes. Reproduced with permission.^[^ ^50^ ^]^ Copyright 2014, Royal Society of Chemistry. f) Simplified flash‐cell modeling, where Wells–Dawson‐type POMs were used for the realization of the floating gate in a non‐volatile molecular memory material. Reproduced with permission.^[^ ^194c^ ^]^ Copyright 2013, John Wiley and Sons. g) Schematics of the fabricated panel where a passivated POM layer is sandwiched between two Ta_2_O_5_ films. Reproduced with permission.^[^ ^213^ ^]^ Copyright 2016, American Chemical Society. h) Conceptual schematics. semiconductor (CMOS)‐compatible long‐term‐retention molecular capacitive cell (POM/Ta_2_O_5_). Reproduced with permission.^[^ ^54^ ^]^ Copyright 2019, John Wiley and Sons. i) Schematic illustration of arrayed and single sandwiched structural device (ITO bottom electrode/PW/PAH/GO/Al top electrode). Reproduced with permission.^[^ ^214^ ^]^ Copyright 2018, John Wiley and Sons. j) Schematic illustration of an RRAM device (left) and a high‐resolution TEM image (right) of its cross‐section, reveal that a POM‐based composite is sandwiched between Pt and ITO electrodes (ITO/PW@PMMA/Au). Reproduced with permission.^[^ ^34^ ^]^ Copyright 2019, Royal Society of Chemistry. k) Schematic of a network with the SWNT/POM complex network, where yellow cuboids, black tubes, and purple spheres represent the terminal electrodes, SWNTs, and POM particles, respectively. Reproduced with permission.^[^ ^41^ ^]^ Copyright 2018, Springer Nature. l) Full circuit schematic shows that the application of a sine wave (of 11 Hz, ±1 V) at the yellow‐colored electrode pad connected to the SWNT (black line)/Por–POM film (green circle), produces outputs from different electrode pads. A function generator is used where the output from one pole is fed as the input signal, while the other pole is grounded via a 50 Ω resistor (z value, orange box). Subsequently, all outputs are taken from the DAQ system (which is also grounded via a similar 50 Ω resistor) to complete the full circuitry. Reproduced with permission.^[^ ^215^ ^]^ Copyright 2022, John Wiley & Sons. m) Conceptual illustration. “Metal−DNA‐origami−POV6” heterostructure permitting the application of memristive functions via multi‐logic functions because of POV6 (violet) and a synaptic dynamic due to an interplay between the Au (dark yellow), DNA‐origami (blue), and counter ions (green). Reproduced with permission.^[^ ^59^ ^]^ Copyright 2023, American Chemical Society.

In a molecular junction (MJ), molecules are placed between the electrodes and a voltage is applied, as a result, electrons are injected into the molecules from one electrode and collected at the other electrode.^[^ ^210^ ^]^ Thus, measured electrical properties are strongly influenced with the number of layers or packing‐density of molecules. Preferably, a weak electronic coupling between the electrodes and molecules is mandatory to achieve a long data retention time (low electron transfer rate) of the memory. With a weak molecule‐electrode coupling, the electronic structure of the molecules is not strongly perturbed in the solid‐state MJ, compared to that in solution, and thus, the electron transport properties emerged from the MJs are mainly related to electronic structures of the molecules.^[^ ^210a^ ^]^ A weak electronic coupling between the POMs and the electrodes, highlighting the applications of redox‐active POM for molecular memory.^[^ ^210a^ ^]^ **Figure** **9**a shows a vanadium‐POM molecule sandwiched between the Au and Pt electrodes (Au‐POM‐Pt), which has the ability to exhibit multiple resistive states.^[^ ^52^ ^]^

In POM‐based RRAM and capacitive memories, where POMs contact a metal electrode, the high electron injection performance can be attained by decreasing the electron injection barrier, a difference between with positions of LUMO level of the POMs and the *E*_F_ of the metal electrode. The position of LUMO level of molecular POMs is usually below the *E*_F_ of metal electrode (Figure 9b is showing LUMO of various POMs with respect to *E*_F_ of Al‐electrode) and hence, reduction of POM, with electron transfer from the metal electrode to POMs, is energetically a favorable step. The first programming operation, the programming voltage is closely related to the LUMO level of the POMs. Therefore, the POM cluster could result in a great decrease in the programming bias, which is originated from the small electron injection/extraction barrier. It has been reported that incorporation of POM can bring the LUMO of the host (organic) matrix to match well with the LUMO of POM. As a result, electrons from the metal electrode cause reduction of POMs, and then flow from the LUMO level of POM to that of organic motif. Thus, the interfacial modification with POM‐interlayer, creates an Ohmic contact which promotes overall electron transport. The intriguing capabilities of redox‐active POMs to behave as electronsponge, advocates the versatile potential of POMs in designing next‐generation memory devices.^[^ ^75^ ^]^

It has been reported that the switching behavior of nanoelectronics is largely related to the ratio of donor‐acceptor species, where donors increase the HOMO level, while acceptors decrease the LUMO level. Thus, the incorporation of strongly electron‐withdrawing acceptor groups increases the energy barrier between electrodes and switching materials, which results in a higher turn‐on voltage. Thus, the hybridization of active dopants (metal NPs and metal oxide NPs) with the organic matrix, modulating the charge injection barrier and ions transport properties, is a valuable strategy to control the CFs towards improving the electronic performance.^[^ ^211^ ^]^ Figure 9c shows a supramolecular assembly strategy for POM clusters to realize charge trapping and CF formation in low‐power NVMs for neuromorphic computing. Downscaling of integrated memories (PC main memory, USB memory, and SSD memory) to the sub‐10 nm level^[^ ^212^ ^]^ permits the integration of stimuli‐responsive molecules, with 3D structural frameworks and discrete energy levels, as switching elements. The tris(alkoxo)ligated Lindqvist‐type hexavanadate compounds [V^V^_6_O_13_(OCH_2_)_3_CR_2_]^2–^ (or POV6) show a compact octahedron‐like structure composed of six vanadium (3d) addenda atoms in their highest oxidized state (V^5+^). These POV6 structures exhibit a low molecular charge (2–) and high stability. POV6 can store multiple different, potential‐induced logic states at room temperature, serving as physical multi‐state switches. Under the influence of an external electric potential, by the metallic STM tip, the electronic structure of surface‐confined molecular POV6 allows single electrons transfer from the STM tip to the POV6 core, which leads to stepwise reduction of V^V^ to V^IV^ (Figure 9d), where decrease in the electron tunneling resistance causes a corresponding increase in the electronic conductance, suggesting a molecular memristive behavior. Subsequently, the observed potential‐induced change in the conductance of molecules signifies the suitability of POMs for resistive memory applications.^[^ ^74^ ^]^ Additionally, the uptake electrons also enhance the electronic charge of these organically functionalized POV6. Thus, the observed charge storage feature also suggests POM's prospects towards charge‐based memory devices as molecular capacitors.^[^ ^205^ ^]^ In both cases, the unique electron‐sponge nature of POV6 makes POMs a highly appealing and worthwhile electronic component. Thus, this solution‐processed technique pushes the development of advanced electronic devices for neuromorphic computing and biotechnology.^[^ ^74^ ^]^

A hybrid polymer of Anderson‐type POM ([N(C_4_H_9_)_4_]_3_[MnMo_6_O_18_[(OCH_2_)_3_CNH_2_]_2_]) and ethyl methacrylate (MMA), has been reported as multilevel resistive switching memory (Figure 9e), to prove that POMs can preserve their frameworks even after one or more reversible loss or gain of electrons.^[^ ^204^, ^206^ ^]^ As POMs have the ability to exhibit multiple discrete redox states in a narrow potential range confirming them as suitable materials to design a high‐performance rewriteable multilevel resistive memory.^[^ ^50^ ^]^ Evidencing that POMs can behave as charge‐trapping elements, fulfilling a primary requirement to develop novel NVMs.

Other than the most explored Keggin­POMs, Dawson­type core–shell POM molecules ([W_18_O_54_(SeO_3_)_2_]^4−^) have been utilized as storage nodes for MOS flash memories (Figure 9f). Theoretical investigations have revealed that the device performance largely depends on the position of the POM‐molecules integrated into the storage media. Moreover, the authors presented a multilevel, multi‐scaled computational framework to simulate flash memory devices. Comparative analysis of the electrical behavior of the core–shell POMs with central cores as [(Se^IV^O_3_)_2_]^4−^ (where selenium exists as Se^4+^) and [Se^V^_2_O_6_]^2−^ (selenium exists as Se^5+^) suggests that the emergence of higher oxidation state could be used to design a novel “write­once–erase” Memory. This work shows that the electrical features of POMs are mainly dependent on the configuration of a core that resides in the shell of a POM‐framework.^[^ ^216^ ^]^

POM‐based hybrid materials exhibit great potential to perform as storage nodes for molecular nonvolatile flash memories owing to their very promising memory and retention characteristics. POM‐fabricated memory cells demonstrate attractive features, including a high density of trapping sites owing to the stable 3D framework of POMs, electronic interaction with silicon, ease of direct accessibility of electronic levels, and inherent retention of information under room temperature. Thus, there is great potential for POMs in the development of advanced molecule‐based storage and memory devices. Electrostatically self‐assembled Keggin‐type tungsten‐POM on the surface of Ta_2_O_5_ matrix, high surface‐to‐volume ratio transition metal oxides, has constructed a composite material (Figure 9g) that revealed remarkable compatibility with CMOS technologies together with outstanding potential for electron trapping as well as retention features.^[^ ^213^ ^]^ This work demonstrated POMs' versatile nature and their scope in designing molecular nonvolatile flash memory arrays.

POMs hyperstructures behave as electronically tunable components in advanced electronic devices and therefore exhibit great potential as charging nodes of tunable charging level for molecular memories owing to POM's ability to tune their HOMO–LUMO level gaps, and electron localization length at room temperature. It has been observed that a POM nanocluster on SiO_2_ can offer current modulation, thus size‐dependent current regulation through single electron tunneling is attributed to the electronic structure of the molecular clusters. The ability of POM to exhibit self‐assembly as well as tunable electronic properties determine their credibility for on‐chip molecular electronics operative at room temperature. POM's ability to transport and confine charge authenticates them as prime candidates for nanocircuitry. Moreover, the structures realized this way have properties particularly stable in ambient conditions. They are, thus, promising for solution‐printed molecular semiconductor films with electronic properties of noticeable tunability, high stability, and predictability. Electrical measurements of POMs under real device (i.e., ambient, examined on SiO_2_ platforms) conditions reveal the reliability of tunable electronic properties of POM structures. This is likely to boost their demand for a variety of potential applications, rendering them a smart material to develop novel devices.^[^ ^217^ ^]^ A joint “solid‐solution‐vapor deposition” approach has been reported to develop the first‐documented CMOS‐compatible long‐term‐retention memory cell, consisting of a stack of functional metal oxides around a Ta_2_O_5_/[PW_12_O_40_]^3−^ core (Figure 9h). This new fabrication technique has proved very helpful in alleviating the main issues related to the fabrications in the field of molecular electronics. POMs integrated cell reveals a high >7 V memory window and write–read times down to 10 ns, capable of high‐speed storage. In this composite, each memory element consists of a three‐dimensional assembly of molecules up to the densities of 6.8 × 1014 cm^−2^ storage nodes (i.e., individual molecular POMs) per monolayer that can store charge accounting to 109–327 µC cm^−2^ for single POM films and 258–774 µC cm^−2^ for 3D Ta_2_O_5_/[PW_12_O_40_]^3−^ core. Thus, this system has charge density that is a multiple of a typical flash memory (≈2 µC cm^−2^). POM/Al_2_O_3_ cells reveal a maximum of 37% boost on information density storage with the fulfillment of the current commercial threshold of 10 years of data‐retention time at ambient conditions. It blazes the trail for a large number of POM‐based NVM products.^[^ ^55^ ^]^

An atomically thin layer of sp^2^ hybridized carbon atoms of graphene arranged in a honeycomb lattice exhibits unique 2D electronic transport responsible for originating strong conductivity and has led the development of various 2D materials, particularly graphene oxide (GO) derived materials with potential diverse applications in thin‐film‐based electronic devices.^[^ ^218^ ^]^ Molecular POMs have ability to convert GO into reduced‐graphene oxide (r‐GO) under UV irradiation owing to their photocatalytic properties. The molecular POM not only acts as a photocatalyst during the green photoreduction process, but also can adhere on the surface of r‐GO sheets as an anionic stabilizer to avoid the aggregation of r‐GO sheets.^[^ ^219^ ^]^ Hence, POM offers great potential to develop efficient composite materials with r‐GO, especially r‐GO and POM‐based composite films, r‐GO–POM, can be developed and immobilized on various substrates, including silicon wafers, flexible polymers, and quartz glass. It has been observed that composite film, constructed of Keggin‐type tungsten POM (H_3_PW_12_O_40_) and r‐GO‐based transistors, has the potential to demonstrate ambipolar features and remarkable transport properties for both holes and electrons. The field effect transistor characteristics, including charge carrier mobility and ON/OFF ratios depend on the number of deposited layers and can be controlled with the film thickness.^[^ ^207b^ ^]^ Two types of memory characteristics are, generally, achieved from GO, including the write‐once‐read‐many‐times (WORM) arising from the GO‐based memory and the rewritable bipolar resistive switching derived from the rGO‐based counterpart.^[^ ^214^ ^]^ Incorporation of a POM layer into a GO‐based RRAM has the ability to deliver a configurable memory characteristic between WORM and bipolar resistive switching under UV irradiation (Figure 9i). The resistive switching mechanism is usually related to the behavior of GO, depends on the number of oxygen‐containing groups and the POM's electrons accepting ability, determines interface property between GO (RGO) and POM, which subsequently confirms the nature of charge trapping or de‐trapping mechanism. It demonstrates that the transition between the WORM and bipolar resistive switching is related to the modulation of the POM and GO interface barrier. Thus, it is believed that composites obtained on the incorporation of appropriate POMs into GO or r‐GO can be envisioned as attractive constituents in designing novel molecular memories.^[^ ^86^ ^]^

Molecular memories have ability to perform with few electrons at the molecular level, demonstrate high‐density data storage capability and thus grasp an opportunity for integration into resistive random‐access memory (RRAM) devices to enhance their data storage ability. Inherent rich reversible redox potential of POMs permits them to initiate a resistive switching behavior through a localized redox reaction of POM molecules. Generally, the resistive switching mechanism in POM‐based resistive switching memory is accessed from the formation/rupture of the conductive filaments, owing to the migration of oxygen vacancies in molecular POMs. Redox‐based NVMs, wherein a nanoscale redox reaction occurs to originate resistance change in external electrical stimuli, are highly appealing owing to their great potential to design next‐generation memory and neuromorphic computing systems (Figure 9j). In‐depth understanding of sophisticated POM‐based molecular memory device molecules allows a new step ahead toward realizing electronic devices at a nano‐sized scale.^[^ ^34^ ^]^

Immobilization of POMs onto single‐walled carbon nanotubes (SWNTs) can potentially enhance the electrical conductivity owing to redox active nature of POMs. It has been observed that host–guest redox‐active hybrid of POMs and SWNTs (POM@SWNT) has the ability to conduct charge to and from the guest POM molecules. Thus, immobilization of POM on CNTs facilitates efficient electron transfer throughout the network of composite (Figure 9k). Intimate attachment of molecular charge‐storage sites and conductive hosts is a highly appealing assembly in the development of next‐generation molecular‐electronics, electrocatalysts, and energy‐storage systems.^[^ ^220^ ^]^ In recent years, brain‐inspired computing systems have earned remarkable attention owing to their huge potential to serve as intelligent, robust, and low‐power computations systems.^[^ ^12b^ ^]^ Compare to aluminum hardware, neuromorphic hardware much largely roots to neuroscience, wherein a dense as well as a complex network of spiking neurons is essential to access intelligent abilities.^[^ ^41^ ^]^ However, in the available neuromorphic devices, both the integration density and wiring complexity, reveal intelligent information processing ability, is far less than that of human brains.^[^ ^221^ ^]^ Molecular POMs along with highly dense networking of SWNTs have been explored as molecular‐neuromorphic devices. Experimental evidence demonstrates that SWNT/POM network is a capable system to generate spontaneous spikes and noise. The basic learning ability of the network is exhibited with the introduction of reservoir computing (RC), which uses spiking dynamics and network complexity. This effort reveals that the redox nature of POM can be used to develop complex networks for the development of neuromorphic devices.^[^ ^41^ ^]^ In addition, host−guest complexation of POMs and macrocyclic organic matrices, such as cyclodextrins reveals single memory units to demonstrate multistate‐switching by a change in the molecular resistance which is attributed to the reduction of individual cationic metal centers of POMs.^[^ ^222^ ^]^ Similarly, an organic–inorganic memristor composed of POMs and organic polymer has been used as a reservoir for temporal information processing, where the formation of the metal CFs determined the resistive switching behavior of the device. During this process, the electrostatic adsorption between POMs and Ag cations plays a key role in the fast mobility of cations that is confirmed by the molecular dynamic simulation. Since the POMs remain intact in multiple redox states which is essential for the setting and resetting process, the POM‐based device exhibits enough tolerance for further neural computing applications.^[^ ^58^ ^]^

To address the bottleneck of separate memory, processing units, and high fabrication cost associated with device downscaling, “beyond CMOS technologies” has been evolving equally at the forefront as “more‐than‐Moore” devices. Thus, artificial platforms analogous to human brain‐like capabilities are now becoming a reality with devices based on nonlinear dynamics.^[^ ^172^ ^]^ Recently, one of the most complex colloidal neuromorphic devices has been reported, where single‐walled carbon nanotubes modified with protonated tetraphenyl porphyrin and Keggin‐type polyoxometalate, namely the [H_4_TPP]_2_[SV_2_W_10_O_40_] or (Por–POM) complex (SWNT/Por–POM) integrated with a microelectrode array was used as a physical RC (in‐materio RC) platform (Figure 9l).^[^ ^215^ ^]^ It has been reported that a thin layer of redox‐active Por–POM on metal electrodes demonstrates a negative differential resistance (NDR) and pinched hysteresis loops, owing to their charge transfer redox‐mediated capacitive nature.^[^ ^223^ ^]^ The influence of a simple sine wave to one randomly selected input causes a series of complex outputs, which is due to the NDR behavior of Por‐POM and a complex, randomly oriented, and interpenetrated network of SWCNTs. Hence, transforming a single into a set of time series of the character, is a signature of brain‐like computation, which is attributed to the intrinsic property of the material. It also shows the scale‐free nature of electrical processes in the network, therefore, the device potential is considered as an RC system. In order to verify this hypothesis, the device was used to generate an arbitrary waveform by computing linear combinations of signals from various different outputs, which was performed with software support and the weights for operations were computed with an artificial neural network, which can be considered as a digital output layer of the reservoir.^[^ ^215^, ^224^ ^]^

Recently, a bio‐hybrid heterojunction, in a hybrid of DNA‐augmented Lindqvist‐type polyoxovanadate (POV6) complex with the six‐helix bundle (6HB) DNA origami carrier, prepared via a solution‐processed assembling technique, has been reported to exhibit switching characteristics (Figure 9m). In this design, DNA‐origami immobilized on the gold (Au) surface via thiolate groups, serves as a carrier (ad‐layer) structure to ensure a controlled hybridization of the 6HB with DNA‐augmented tris(alkoxo)‐ligated POV6 units. In this design, stepwise electron acceptance of DNA‐confined POV6 units demonstrates a multi‐logic function. The developed molecular‐device can be addressed and read out at the nanoscale via an STM tip. Investigations have revealed that potential on the nanoscale STM tip, oxidation state of POV6, and the mechanism of interaction of POV6 countercations, determine the electron acceptance/injection into the originally non‐conducting DNA and the consequent release to the Au(111) substrate. Experimental and theoretical studies show that POM‐incorporated bio‐hybrid heterojunction has huge potential to develop biocompatible artificial synaptic devices.^[^ ^60^ ^]^

#### 5.6.1 Theoretical Studies of POM‐Based Memory Devices

**Figure 10:**
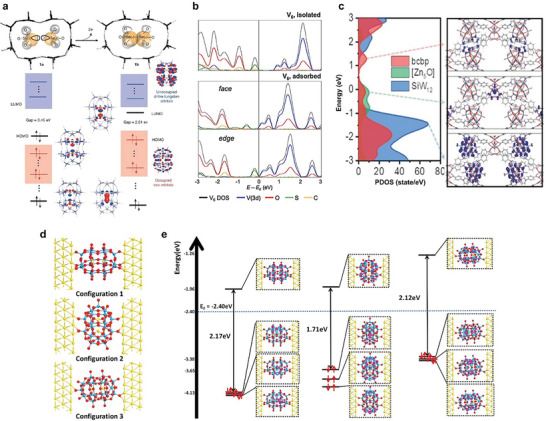
Computational studies of POM‐based memory devices. a) The formation of Se(V)−Se(V) bond within the [W_18_O_54_(SeO_n_)_2_]^4−^ cage. Reproduced with permission.^[^ ^80^ ^]^ Copyright 2021, John Wiley and Sons. b) DOS and projected DOS (PDOS) for isolated (top), and adsorbed (on Au(111) in the face‐ and edge orientation) (bottom) V_6_ (a fully oxidized Lindqvist‐type POM [V^V^_6_O_13_(OCH_2_)_3_CCH_2_SR_2_]^2−^ (R = Me). Reproduced with permission.^[^ ^173^ ^]^ Copyright 2021, American Chemical Society. c) PDOS and electron‐density distributions of the FMO of SiW12@Zn‐bcbp. Reproduced with permission.^[^ ^227^ ^]^ Copyright 2023, John Wiley and Sons. d) Three Au−POM−Au configurations and e) their relative HOMO−LUMO energy positioning along with their respective frontier Kohn−Sham molecular orbitals (blue dotted line shows *E*_F_). Reproduced with permission.^[^ ^209b^ ^]^ Copyright 2021, American Chemical Society.

Core–shell POMs, Dawson‐like archetypes with general formula [M_18_O_54_(XO_n_)_2_]^m‐^ (where M = Mo or W; X = P, S or Se; n = 3 or 4 and m = 2 to 8), have gained huge attentions as promising candidates to serve as storage nodes for metal–oxide–semiconductor (MOS) flash memories owing to configurable core (heteroatom dopants P, S, or Se), nanoscale size (≈1.2 × 1 × 1 nm), high thermal stability, and most importantly, the ability to trap charges. DFT studies have also highlighted the role of heteroatoms (P, S or Se) of inner cores of the Dawson‐like archetype POM [W_18_O_54_(XO_n_)_2_]^m−^ (X = P, S or Se), which reveal a change in the electronic structure in response to a redox process (**Figure** **10**a). The positions of two inner heteroatoms‐containing cores, very next to each other, within a POM shell allow them to interact via lone‐pairs of electrons. Therefore, the ejection of a pair of electrons from the outer POM shell permits heteroatoms of two inner cores, in case of ([W_18_O_54_(SeO_3_)_2_]^4−^), to develop a Se‐Se bond, owing to change of oxidation state of selenium from Se (IV) to Se (V).^[^ ^195^, ^205^ ^]^ In the case of POM−semiconductor direct contact, the energy gap between the LUMO of POM and conduction band of semiconductor becomes smaller, allows high electron‐injection efficiency which is essential for flash memory devices. Importantly, the hysteresis was observed with a gap of about 0.2 V between the downwards and upwards voltage sweeps, which was related to oxidation process of Se(IV) → Se(V).^[^ ^205^ ^]^ Thus, the capability of heteroatoms of inner cores of POM molecules to achieve next higher oxidation state in response to a redox potential makes the way to design novel nanoscale flash memory.^[^ ^225^ ^]^ Additionally, the multi‐level computational framework analysis has predicted that molecular POMs have ability to perform as a floating gate, highlighting the potential for molecular‐based flash memory cells. The performance of a memory device is primarily assessed by the number of molecules in the storage media.^[^ ^205^ ^]^ The high data storage density of multilevel memories can be achieved with the increase in the number of device cells, while a limited increase in the storage density can be attained via device size shrinking or with the multilayer stacking structures approach. Consequently, compared with scaling down as well as stacking structure approach, application of multilevel data storage materials is a most attractive strategy to achieve high‐density memory devices.^[^ ^226^ ^]^

The computational studies on molecular POMs, polyoxovanadates, have revealed that the experimentally observed staircase conductivity is mainly related to the oxidation/reduction molecules. The incorporation of additional electrons in the metal‐d band increases POM's ability to transfer electrons from one electrode to the other.^[^ ^228^ ^]^ DOS is a mathematical function that shows the number of electronic states versus the energy, demonstrating the band structure of a periodic system with occupied or valence bands below the *E*_F_ while empty or conduction bands above the *E*_F_. V_6_ (a fully oxidized Lindqvist‐type POM [V^V^_6_O_13_(OCH_2_)_3_CCH_2_SR_2_]^2−^ (R = Me) has a valence band close to *E*_F_ of gold, a large oxo band of the POM at lower energies, and an empty vanadium‐like band with a band gap < 1 eV (Figure 10b). Unlike classical solid‐state compounds used in RRAM cells which possess large band gaps (TiO_2_ = 3 eV; SiO_2_ = 9 eV), POMs have characteristic smaller band gaps which may result in low‐potential working regimes in nanodevices.^[^ ^174^ ^]^ For a fully oxidized V6‐Au(111), a narrow transmission peak was identified in the spectrum only 0.05 eV away from *E*_F_ (Figure 10b, top), predicting that a bias voltage can cause the conduction. Next, in the case of applied voltage, this peak gradually shifts and overlaps the potential window. Furthermore, for a bias potential of 0.6 V, the peak completely lies in the potential window, revealing a strong probability for conductivity. Noticeably, theoretical studies reveal that successive electron additions V6 bring peaks, of transmission spectra of V6−Au, near the *E*_F_ (Figure 10b), which is confirming a gradual increase in the molecular conductivity. Prominently, the changes in the redox‐state of a molecule with an external voltage, create successive conductivity steps. These findings establish a firm foundation that the modification of the redox‐state of molecules under the influence of external applied potential, regulates the molecular conductivity.^[^ ^174^ ^]^

Till to date, numerous RS devices based on information memory and neuromorphic computing (NC) have been developed. Among them, RS‐based optoelectronic synaptic devices have opened new possibilities toward human brain‐like function simulation, logic computing, and multifunctional NC in artificial vision systems.^[^ ^227^ ^]^ The synaptic conductance in these devices is reversibly tuned through optical and electrical signals. Recently, conductive donor‐acceptor hybrid heterostructure, SiW_12_@Zn‐bcbp, was reported as stimuli‐responsive material to modulate the RS behavior of optoelectronic synaptic devices. Figure 10c shows the results of DFT calculation for SiW_12_@Zn‐bcbp, where the PDOS of SiW_12_@Zn‐bcbp demonstrates that [Zn_5_O] dominates the valence band maximum (VBM), whereas electron‐deficient bcbp ligands dominate the conduction band minimum (CBM). Moreover, the electron‐density distributions of the frontier molecular orbital (FMO) have also revealed the electron transfer between the [Zn_5_O] and the bcbp to produce V^•+^ radicals. Thus, it predicts the multielectron transfer in the ternary POM@Zn‐bcbp hybrid. It is well known that under the influence of applied external voltage, the electron‐sponge nature of POM induces RS behavior in POM‐based hybrids which is due to multielectron uptake from the contacting metal electrode.^[^ ^229^ ^]^ Photoinduced electron transfer generally determines the photo‐induced current in the optoelectronics.^[^ ^227^ ^]^ Under light illumination, more V^•+^ radicals and W^V^ species were produced, accelerated charge separation and transfer, consequentially increasing the photo‐induced current. As a result, abundant electron transfers followed the electron‐flowing route, however, the recombination process of electrons and holes resulted in the decay process. The ternary POM@Zn‐bcbp hybrid material performed as a synaptic device and revealed dual‐modulation of synaptic plasticity owing to the synergetic effect of electron reservoir POM and photoinduced electron transfer. The result provides a facile and effective strategy to customize multi‐modality artificial synapses, which is essential for developing high‐performance neuromorphic devices.^[^ ^227^ ^]^

One of the key challenges in exploring single MJs is the lack of structural details at the molecule level, that is, how the molecules are attached to the electrodes. It is well known that the interaction of the molecule to the electrode contact significantly influences the current flow in molecular electronic systems.^[^ ^230^ ^]^ Thus, to investigate the nature of contact and subsequent influence, three different types of junction‐setups as 1, 2, and 3 have been reported for [W_18_O_54_(SO_3_)_2_]^4−^, where oxygen atoms of redox‐active molecule (POM) contact to gold (Au) electrode in three different orientations (Figure 10d). The binding energies calculated for all three configurations show that the binding energy (eV) values are in positive, and much higher value than the thermal energy which shows a very strong absorption of the molecule to the electrode (Au) surface. Therefore, the energy levels, specifically the HOMO and LUMO calculated for all configurations, and their energetic properties were compared (Figure 10e), confirming that the molecule‐electrode configuration strongly influences the energy levels of the molecule. Figure 10e shows that the HOMO−LUMO gap for all configurations is very close to the value (1.89 eV) of the isolated molecule. Evidently, the alignment of the energy levels to the *E*_F_ is different for each ease. In configurations 1 and 2, the *E*_F_ lies near the HOMO than the LUMO, while for configuration 3 it lies almost halfway between the HOMO and LUMO. This reveals that the high contact strength to the electrodes stabilizes the HOMO level and brings the LUMO near to the Fermi energy. This behavior is associated with the number of bonds between the molecule and the Au‐surface. For configuration 1, there are three contacts (bonds) between the POM and Au‐electrode, which is showing a stronger contact. For configuration 1, the HOMO−2, HOMO−1, and HOMO all lie within 0.02 eV of each other and thus become degenerate states. Also, configuration 3 shows a similar degeneracy, revealing that the “horizontal” orientation pulls these energy levels closer, confirming that the electron density lies on part of the central oxo bands. However, for configuration 2, the densities for these levels occur on the upper and lower oxo bands, and thus these levels are relatively discrete and result in differences in energy.^[^ ^210b^ ^]^ From the design point of view, the HOMO level can be related with the valence band edge (*E*_V_) while the LUMO level with the conduction band edge (*E*_C_). For configurations 2 and 3, the “horizontal” orientation creates larger HOMO−LUMO gaps (2.17 and 2.12 eV) compared with that of the isolated molecule (1.89 eV). In contrast, configuration 2 with a “vertical” orientation shows a smaller HOMO−LUMO gap (1.71 eV). This suggests the orientation of the molecule to the electrode strongly influences HOMO−LUMO gaps. The orientation of the molecule mostly affects the HOMO and near‐HOMO levels, subsequently pulling them closer together and stabilize, in turn, the gap of LUMO widens. This can be anticipated because these energy levels are full of valence electrons which facilitate molecule‐electrode bond formation, where stronger contacts cause higher predicted currents. Therefore, horizontal geometry leads to the most favorable transmission profile for this molecule. The contact geometry and the number of bonds between the redox‐active molecules and the electrodes determine the current flow, which is crucial in designing high‐performance data storage devices.^[^ ^210b^ ^]^

### 5.7 POM‐based Nonvolatile Neuromorphic Devices versus State‐of‐the‐Art Counterparts

Semiconductor quantum dots with spin qubits are considered as one of the imperious routes to realize quantum computers,^[^ ^231^ ^]^ however, the scalability to a higher number of qubits poses a grand challenge. Generally, modifying the electronic structure of TMOs with the incorporation of dopant ions is the most common strategy to modulate the electron density near the *E*_F_. Molecule‐based memory devices rely on quantum states and, hence, charge localization enables multiple charge states.^[^ ^232^ ^]^ Unlike traditional TMO‐based designs, the electron‐sponge nature and resultant self‐assembling capability of molecular POMs develop identical units of localized spins to realize the qubits, rendering POMs as reversibly switchable molecules for the engineering of molecular electronic devices. The leakage current, also regarded as tunneling current, increases with the decreasing thickness of the dielectric layer, causing the device unreliable.^[^ ^213^, ^233^ ^]^ Realizing praiseworthy memory features of large memory window (for the write state) along with high retention without the use of blocking oxide, is highly demanding for NVM devices. Developing an interface with molecular frameworks in the localized dipoles of a cell, influences the band alignment in memory devices. Compared with the state‐of‐the‐art materials including 2D species,^[^ ^234^ ^]^ POMs which incorporate a variety of redox‐active metals in their 3D frameworks, offer better modulation of the electronic structures, which is highly desirable to achieve appropriate charge flow. Unlike classical solid‐state compounds used in RRAM cells which possess large band gaps (TiO_2_ = 3 eV; SiO_2_ = 9 eV), POMs have characteristic smaller band gaps which may result in low‐potential working regimes in nanodevices.^[^ ^174^ ^]^ Redox‐active POM clusters can also serve as ideal materials to develop new types of nanoelectronics with conventional semiconductors, where the direct contact of POMs with the semiconductor layer, can substantially decrease the energy difference between the conduction band of the semiconductor and the LUMO of POMs, suggesting a high electron‐injection efficiency for the memory devices. POMs, compatible with CMOS technologies owing to high solution‐processability,^[^ ^60^ ^]^ serve as a memory cell owing to electron‐sponge capabilities and therefore, unlike traditional TMOs based memories, reveal promising memory and retention characteristics for molecular nonvolatile multi‐level switches and flash memories.^[^ ^213^ ^]^ In artificial intelligent (AI) systems, deep learning which originates from the artificial neural networks (ANNs) of classical von Neumann computing leads to undesired power consumption. On contrary, brain‐inspired framework of physical RC that offers fast learning with low‐cost training, has the ability to perform computation using high‐dimensional, nonlinear, dynamic substrates. In this scenario, a memristor, owing to its nonlinear features and memory behavior, emerges as a simple, adaptable, and efficient framework to construct physical RC to exhibit memristor‐implemented ANNs toward neuromorphic computing. Therefore, unlike classical von Neumann computing, POM‐based RC has displayed increasing popularity toward neuromorphic computing.^[^ ^211^ ^]^

## 6 Outlook and Future perspectives

Polyoxometalates (POMs) is a unique class of redox‐active metal oxide clusters that have a variety of structures and applications. This review briefly describes POMs as superior contender materials, owing to their exclusively unique features such as electron sponges‐like nature, to design next‐generation molecular devices. Valuably, the mandatory features of a contender material that make it suitable for energy conversion and nonvolatile neuromorphic devices, are systematically discussed. Moreover, the recent progress of POMs in the fields of electrocatalytic OER/HER and nonvolatile neuromorphic devices are also discussed.

The realization of POM‐based molecular devices can open a corridor to enhance the device performance owing to the availability of multiple redox states in resultant devices. The fascinating properties of POMs are associated with plenteous active sites of POMs, which are invaluable for energy‐related technologies and electronic information storage devices.

The application of POMs as electrocatalysts is deliberated for both OER and HER processes. Regarding water‐splitting electrocatalysts, the following principles provide a firm standing to design robust water‐splitting electrocatalysts: 1) Obtaining precious metals‐free TM‐based materials, POMs, to exhibit multielectron transfers and stabilize high oxidation states to decrease the overpotentials. 2) Conducting DFT studies and then applying corresponding modification experimentally on a material to achieve: i) high selectivity for the targeted intermediate species to suppress the competitive reactions, ii) improved stability of structural frameworks to endure long‐term electrochemical under harsh conditions, and iii) high electrocatalytic activity at small overpotential. 3) Systematically studying the electronic and steric effects of metal ligands. 4) Developing scalable synthesis routes with earth‐abundant elements.

It has been observed that although significant advances have been made in the field of NVM technology; but still more efforts are needed to resolve numerous shortcomings associated with current memory devices such as high‐power consumption, low resistance ratio, variability issues, cell and device level reliability, issues associated with material's deposition and subsequently inappropriate contact with electrodes and thus poor CMOS compatibility, lack of advanced and smooth processing of memory devices.

As is known that the electrical properties of a material are dependent on the number of layers or packing‐density between the electrodes, regarded as molecular junction. Preferably, a weak electronic coupling between the electrodes and molecules is mandatory to achieve a long data retention time (low electron transfer rate) of the memory.^[^ ^210^ ^]^ Thus, new advances are required to overcome the grand challenge of electrical contact of redox‐active molecules with the metal electrodes, which is imperious to realize high‐performance nonvolatile logic‐in‐memory and neuromorphic computing.^[^ ^74^ ^]^ The designing of neuromorphic devices at the nanoscale (below 10 nm), typically necessitates the incorporation of stimuli‐responsive molecules with 3D structural frameworks to exhibit discrete energy‐levels as active switching components. However, the poor chemical and mechanical stability, specific working conditions (e.g., low temperatures), and the gigantic challenge of how to deposit and contact molecular components in the CMOS devices, are strictly hampering the applications of molecular switching components in real‐world devices.^[^ ^74^ ^]^ POMs have capabilities to develop optimum contact with the electrode and thus empower CMOS‐compatible interfaces.^[^ ^60^ ^]^ On the basis of research advances, it can be concluded that POMs possess distinct architectural features of processability, tunable bandgap, excellent flexibility, and so on. Besides, POMs significantly prohibit the variation in the resistive switching behavior, which commonly emerges under harsh environments of high temperature and humidity, and thus POM‐based device facilitates the realization of high‐density data storage molecular devices.^[^ ^179^ ^]^ Therefore, POMs‐based RRAM devices have revealed significant breakthroughs, owing to fast‐switching behavior, high‐density information storage, relatively low energy consumption, solution‐processable integration for multi‐level switching,^[^ ^60^ ^]^ sufficient endurance cycles, and longer retention time, compared to traditional oxides‐based memories. Al systems rely on deep learning which, in turn, originates from the ANNs. Unfortunately, ANNs depend on the classical von Neumann computing which leads to unnecessary power consumption.^[^ ^211^ ^]^ In contrast, neuromorphic computing, owing to the integration of highly parallelized computing architectures, enables intelligent systems to simulate neurobiology at the nanodevice level with lower power consumption. RC has been known as an alternative brain‐inspired framework for fast learning with low training costs. Memristor appears as an adaptable, simple, and efficient framework for constructing physical RC because memristors can demonstrate nonlinear properties and memory behavior. Therefore, POM‐based RC displays increasing popularity in neuromorphic computing.^[^ ^211^ ^]^

Various POM‐based artificial synapses and neuromorphic computing systems have been established to defeat the bottleneck of Von Neumann's architecture. However, for POMs, there are still some major limitations and challenges to be solved: a) Despite the enhanced flexibility of POMs in the presence of organic cations, the humidity and thermal instability may cause severe damage to the device performance. b) Most of the research efforts focus on the requirements of theoretical study, while a set of certified standards is required to offer a fair assessment of these fabricated devices. c) Facile solution‐processable strategies are needed to anchor molecular components in the CMOS devices, in order to accelerate the applications of molecular switches in real‐world devices.^[^ ^74^ ^]^ In addition, more efforts are needed to investigate the compatibility of lab‐produced devices with CMOS. In other words, further applications of POM‐based RRAM devices are anticipated. Although POM‐based devices have revealed RS phenomena under extreme conditions (such as high temperature or humidity), more suitable hybrids of POMs are required to achieve RS behavior at about 200 °C for nonvolatile neuromorphic devices.^[^ ^179^ ^]^ e) The POMs‐based devices still face serious issues of stability which need to be addressed.^[^ ^191^ ^]^ To overcome the associated shortcomings with the ANN of Von Neumann architectures, POM‐based physical RC has gained much attentions.^[^ ^58^ ^]^ f) Noticeably, despite a wide variety of POM architectures such as sandwich‐type POMs with variety of central cores (Figure 4), most of the explored compositions are relying on Keggin‐type POMs which have only 12‐addenda atoms with a central core of heteroatom (Figure 1).^[^ ^58^ ^]^ Unlike Keggin‐type POMs, sandwich‐type POMs or other POM architectures where central core is encapsulated with 3 or 4 lacunary units, with high number of redox‐active centers in their frameworks which can improve the electron dislocation, can potentially offer high‐performance in electrocatalytic and nonvolatile neuromorphic devices, owing to the improved electron transport properties.^[^ ^7b^ ^]^ Therefore, investigations on a variety of POM frameworks are required to design highly active and durable energy conversion/storage and ultrahigh‐density data storage devices. g) POM‐based materials have witnessed considerable advances toward electrode materials for energy technologies, nonvolatile memory devices, and other nanoelectronics, owing to the electron‐sponge nature of POMs and the abundance of diffusion channels for metal cations (such as Na^+^) in their 3D structural framework.^[^ ^235^ ^]^ Despite significant advances in the fields of POM‐based materials, there are still some challenges (such as (i) low conductivity that needs a conductive substrate to enhance the electron transport properties,^[^ ^236^ ^]^ (ii) inappropriate electronic structure that needs heteroatoms doping for the modulation of d‐band center,^[^ ^33a^ ^]^ (iii) involved ambiguous mechanisms and intricate morphology control) that need to be further addresses.^[^ ^216^ ^]^ Therefore, in‐situ characterizations are needed to investigate the dynamically structural changes of POM‐based materials to uncover the real reaction mechanisms. As POMs are viewed as water‐soluble anions, so their immobilization to desired substrates needs to be handled carefully.^[^ ^25^ ^]^

Overall, continual progress into POMs for energy devices and molecular nanoelectronics (memristors) can be expected to make a remarkable influence toward resolving next‐generation energy and digital information‐related issues, which indispensable to address the challenges of future‐energy, big‐data, and post‐Moore age.

## Conflict of Interest

The authors declare no conflict of interest.

## Author Contributions

All authors researched data for the article. W.A., N.A., K. W., S.A., B.‐B. Y., P.Z., W.Y., M.L., and G.‐H. L. contributed substantially to the discussion of the content. W.A., N.A., and S.A. wrote the article. All authors reviewed the manuscript before submission.
